# Magnetic forces enable controlled drug delivery by disrupting endothelial cell-cell junctions

**DOI:** 10.1038/ncomms15594

**Published:** 2017-06-08

**Authors:** Yongzhi Qiu, Sheng Tong, Linlin Zhang, Yumiko Sakurai, David R. Myers, Lin Hong, Wilbur A. Lam, Gang Bao

**Affiliations:** 1 Wallace H. Coulter Department of Biomedical Engineering, Georgia Institute of Technology and Emory University, Atlanta, Georgia 30332, USA; 2 Division of Pediatric Hematology/Oncology, Department of Pediatrics, Aflac Cancer and Blood Disorders Center of Children's Healthcare of Atlanta, Emory University School of Medicine, Atlanta, Georgia 30332, USA; 3 Winship Cancer Institute of Emory University, Atlanta, Georgia 30332, USA; 4 Department of Bioengineering, Rice University, Houston, Texas 77005, USA

## Abstract

The vascular endothelium presents a major transport barrier to drug delivery by only allowing selective extravasation of solutes and small molecules. Therefore, enhancing drug transport across the endothelial barrier has to rely on leaky vessels arising from disease states such as pathological angiogenesis and inflammatory response. Here we show that the permeability of vascular endothelium can be increased using an external magnetic field to temporarily disrupt endothelial adherens junctions through internalized iron oxide nanoparticles, activating the paracellular transport pathway and facilitating the local extravasation of circulating substances. This approach provides a physically controlled drug delivery method harnessing the biology of endothelial adherens junction and opens a new avenue for drug delivery in a broad range of biomedical research and therapeutic applications.

## Abstract (web summary)

The transportation of large molecules through the vascular endothelium presents a major challenge for *in vivo* drug delivery. Here, the authors demonstrate the potential of using external magnetic fields and magnetic nanoparticles to enhance the local extravasation of circulating large molecules.

The circulatory system provides an efficient route for the distribution of blood-borne substances throughout the body. However, the vascular endothelium, which lies at the interface between the blood and the tissue space, creates a transport barrier for pharmacologic agents by blocking the extravasation of any particulates larger than serum albumin molecules (66 kDa)^1^. Delivery of large drug molecules and drug carriers, including therapeutic proteins, nucleic acids and nanomaterials, occurs either only at the level of the vascular endothelium itself or when the permeability of the endothelial barrier is significantly increased. In this regard, current drug delivery strategies are primarily aimed at treating solid tumours, in which large pores are opened along the endothelium of angiogenic vessels^2^,^3^. Nevertheless, the success of delivering anticancer drugs is often impeded by the inherent heterogeneity in vascular permeability due to the complex interplay between the vessels and the tumour microenvironment^4^. In many other diseases, such as cardiovascular diseases and central nervous system diseases, the vascular endothelium maintains its structural and functional integrity, preventing sufficient drug delivery into the tissue space. Circumventing endothelial barrier is, therefore, the key for achieving an adequate, uniform and targeted distribution of therapeutic agents in many critical disease treatments.

Transport across the vascular endothelium can occur via either transcellular or paracellular route. Both are tightly regulated through a complex network of signalling pathways to maintain the homeostasis of tissue microenvironment^1^. These transport pathways have been utilized for developing efficient biological and biophysical delivery approaches. For example, antibodies targeting aminopeptidase P can trigger caveolae-mediated transcytosis in the lung endothelium, thus promoting cargo transport from blood into lung tissue^5^. Infusion or superfusion of vascular endothelial growth factor (VEGF) can increase vascular permeability and promote transvascular transport of macromolecules and nanoparticles into tumour tissue^6^,^7^. The effect of VEGF on vascular permeability is partly attributed to its regulation of endothelial junctional proteins that control paracellular transport^8^. Alternatively, intracarotid infusion of hyperosmotic agents or vessel dilators is used to transiently open the blood–brain barrier by inducing endothelial cell shrinkage and vascular dilatation^9^. These approaches, however, lack the capability of targeting specific anatomic regions, which can lead to non-specific drug uptake and consequential adverse off-target effects. More recently, attempts have been made to use focused ultrasound and circulating microbubbles to generate localized enhancement of vascular permeability^10^,^11^. However, several issues still remain to be addressed in this approach, including tissue heating, attenuation of ultrasound by complex anatomical structures and the rapid clearance of microbubbles by the mononuclear phagocyte system^12^. There is, therefore, an urgent need for a more efficient target delivery approach in many disease treatments.

Over the past few years, magnetic nanoparticles (MNPs) have emerged as promising drug carriers in image-guided drug delivery, magnetic drug delivery, and synergistic chemotherapy and hyperthermia^13^,^14^,^15^,^16^,^17^. Extensive studies have been performed to increase the internalization/accumulation of MNPs *in vitro* or *in vivo* with an externally applied magnetic field^18^,^19^,^20^,^21^. Moreover, as nano-sized magnets, MNPs have been utilized to deliver mechanical cues to living cells at the molecular level, which provides an unprecedented capability for controlling cell signalling pathways and intracellular events^22^,^23^,^24^,^25^. In our previous studies, we have shown that magnetic force can enhance the endocytosis of MNPs by various cell types including human umbilical vein endothelial cells (HUVECs) without affecting cell viability^20^,^21^. Also, in static culture, the magnetic force applied to intracellular MNPs can modulate the F-actin dynamics and organization in endothelial cells^21^. It is known that VE-cadherin, the key component of endothelial adherens junctions that mediate the paracellular transport across the vascular endothelium, anchors to the actin cytoskeleton in integral endothelium, and this association between VE-cadherin and F-actin tightly controls endothelial barrier function^26^,^27^. Alterations in actin filament organization induced by either actin-depolymerizing or -stabilizing substances can disrupt adherens junctions and increase vascular permeability^28^,^29^.

In this work, we investigate the intracellular magnetic force-induced structural and functional changes of the vascular endothelial barrier through *in vitro* studies using endothelialized microfluidic channels, and *in vivo* experiments with a mouse lateral tail vein model. We demonstrate that vascular permeability in a tissue can be increased magnetically through a two-step process using external magnets—targeting of MNPs to the vascular endothelium and disrupting the endothelial adherens junctions via intracellular magnetic forces from MNPs. This approach provides a physically controlled drug delivery method that opens a new avenue for drug delivery in a broad range of biomedical research and therapeutic applications.

## Results

### MNPs and magnetic force

**Figure 1:**
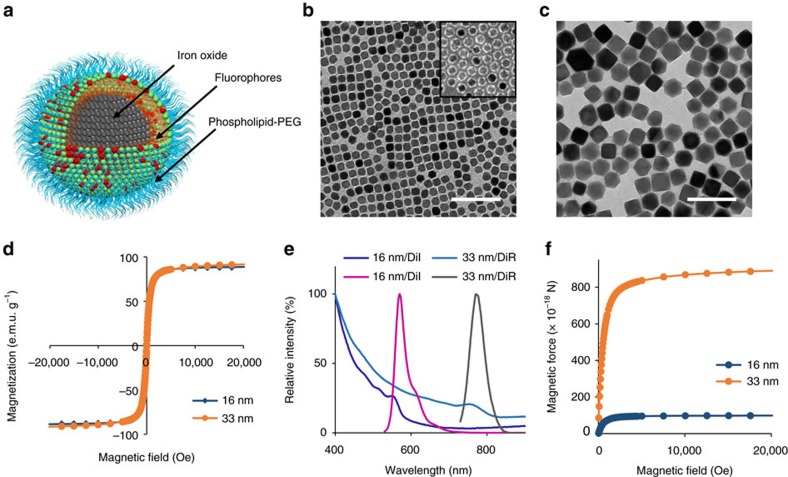
Properties of magnetic iron oxide nanoparticles (MNPs). (**a**) A schematic diagram of MNP. Each MNP contains a magnetite nanocrystal and a phospholipid–PEG coating. The phospholipid layer provides anchoring sites for lipophilic fluorophores such as DiI or DiR for fluorescence tracing of MNP. (**b**,**c**) TEM images of 16 and 33 nm diameter magnetite nanocrystals. Inset in **b** shows phospholipid–PEG-coated MNPs negatively stained with phosphotungstic acid. The phospholipid–PEG coating of MNPs appears as the white corona surrounding the dark nanocrystals. Scale bar, 100 nm. (**d**) Magnetization curves of MNPs. (**e**) Absorption and emission spectra of 16 nm MNPs labelled with DiI (16 nm/DiI) and 33 nm MNPs labelled with DiR (33 nm/DiR), respectively. (**f**) Calculated magnetic force on individual MNPs as a function of the field strength. The gradient of the magnetic flux density was assumed to be 100 T m^−1^.

Both the magnetic properties of MNPs and the applied magnetic field are essential for targeted cellular internalization of MNPs and exerting adequate intracellular magnetic force. Here we synthesized magnetite nanocrystals (16 and 33 nm in diameter) through thermo-decomposition of iron acetylacetonate (Fig. 1a–c)^30^,^31^. Both nanocrystals exhibited saturation magnetization approaching that of bulk magnetite but little remanence at zero field (Fig. 1d). Water-dispersible MNPs were synthesized by coating the nanocrystals with phospholipid-poly(ethylene glycol) (Fig. 1a, inset in Fig. 1b; Supplementary Table 1)^32^. The phospholipid coating facilitated labelling MNPs with DiI (ex/em=549/565 nm) or DiR (ex/em=710/780 nm) for their visualization *in vitro* and *in vivo*, respectively (Fig. 1e; Supplementary Figs 1 and 2).

The applied magnetic field was created using NdFeB magnets (see Methods section). The spatial distribution of the magnetic field was determined by numerical simulation (Supplementary Methods; Supplementary Table 2; Supplementary Figs 3–5). The field strength is ∼5,000 Oe on the surface of an NdFeB magnet. Assuming a field gradient of 100 T m^−1^, the magnetic force on individual MNPs is on the order of 1 fN and ∼900-fold higher than the gravitational force (Fig. 1f).

**Figure 2:**
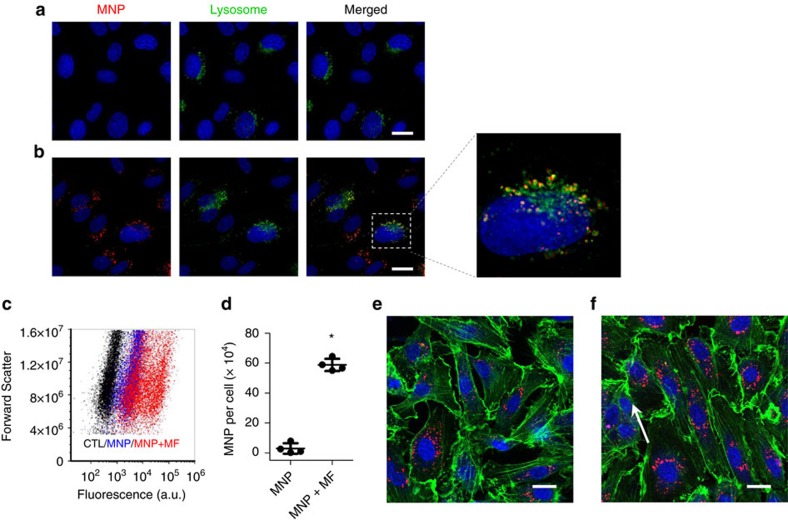
The effect of magnetic force on endothelial cells in static culture. Endothelial cells were incubated with 16 nm MNPs without (**a**) or with (**b**) a magnetic field. Magnetic force increased uptake of MNPs (red) by endothelial cells. Intracellular MNPs co-localized with lysosomes (green). Note that the lysosomes were stained based on virus-delivered transgene expression, which was uneven among transfected cells. Blue, nuclei. (**c**) Flow cytometry analysis of cellular uptake of MNPs without or with a magnetic field. (**d**) Quantification of intracellular MNPs by iron content (mean±s.d.; *n*=4; *P<0.05). Endothelial cells containing MNPs were further incubated without (**e**) or with (**f**) a magnetic field generated by an N52-grade rare-earth magnet (*W* × *H* × *L*=1/2″ × 1/2″ × 1″), respectively. (**e**) The stress fibres (green) of endothelial cells have a random orientation after internalizing MNPs (red). (**f**) After exposed to external magnetic force, stress fibres appear to align with the force (the arrow indicates the direction of magnetic force). Scale bar, 20 μm.

We then examined the magnetic force-regulated MNPs uptake and actin dynamics in static cell culture. As shown in Fig. 2a,b, the magnetic field markedly enhanced the uptake of MNPs by endothelial cells. Intracellular MNPs accumulated primarily in perinuclear lysosomes. The amount of intracellular MNPs shows large variation among endothelial cells, owing to the heterogeneous magnetic force field used in cell culture (Fig. 2c; Supplementary Fig. 3). On the basis of the iron content measurement, these cells contained 5.8±0.7 × 10^5^ MNPs per cell (Fig. 2d). When the endothelial cells with MNPs were further incubated at the edge of an NdFeB block magnet, the total intracellular magnetic force parallel to the cell surface is on the order of 2 × 10^−10^ N. After 1 h incubation, randomly distributed F-actin fibres in endothelial cells became aligned in the direction of the magnetic force (Fig. 2e,f). It is noteworthy that the total intracellular magnetic force is similar to the shear force experienced by the cells at a shear stress of 5 dyn cm^−2^, assuming the surface area of an endothelial cell is 400 μm^2^. It suggests that the MNPs and the magnets used in this study can generate an intracellular magnetic force with the same order of magnitude compared to the shear force on the vascular endothelium in capillaries and post-capillary venules at the surface of the body^33^.

### Endothelialized microfluidic channels

**Figure 3:**
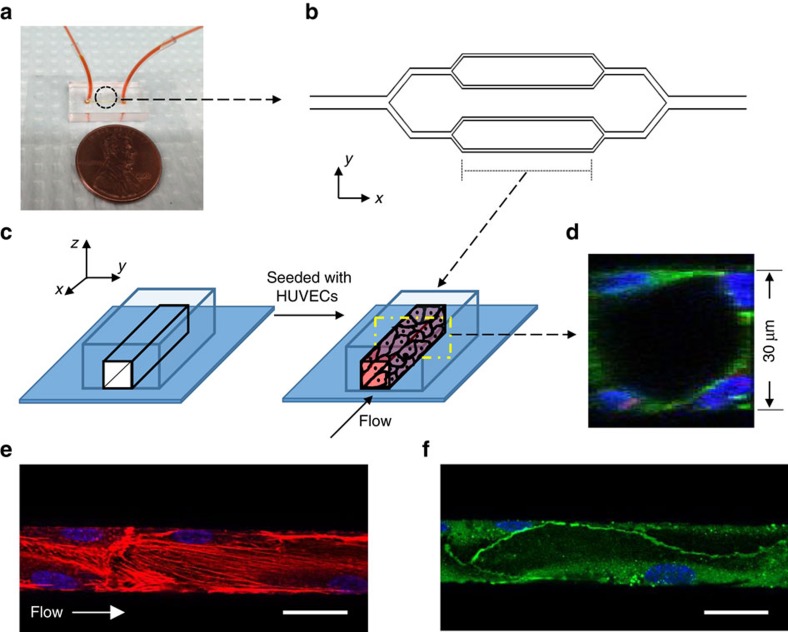
Engineered *in vitro* endothelialized microfluidic channels. (**a**) Macroscopic view of a PDMS microdevice filled with red food colour. (**b**) CAD-based geometric pattern of the microfluidic system. The smallest channels in this pattern are 30 μm in width, and the height of the channels after fabrication is also 30 μm. (**c**) A schematic diagram showing the process of ‘endothelization' of the microfluidic channel. The endothelialized channel is maintained under constant, physiologic flow conditions. (**d**) A representative confocal image of the cross-section of the smallest channel after endothelial cells reach confluency (blue, nuclei; green, actin). (**e**) In the engineered endothelium, F-actin fibres (red) were localized at the cell–cell interface and stress fibres (red) are aligned with the flow (blue, nuclei). (**f**) The engineered endothelium comprises continuous adherens junctions, as shown on anti-VE-cadherin staining (green). Scale bar, 30 μm.

Endothelial cells can mechanosense the fluid shear stress exerted on the cell membrane and respond by organizing actin filaments along the flow direction^34^,^35^. The direction of the intracellular magnetic force relative to the shear stress is, therefore, likely to affect the organization and alignment of preformed actin filaments. We constructed an endothelialized microfluidic system, which not only provided a tightly controlled flow environment mimicking the physiologic hemodynamic conditions (1–10 dyn cm^−2^) in blood vessels but also facilitated microscopic examination of the endothelium (Fig. 3a,b)^36^,^37^. A confluent monolayer of endothelial cells covering the entire three-dimensional inner surface of microfluidic channels was cultured under physiologic flow conditions (Fig. 3c,d; Supplementary Fig. 6). Similar to the vascular endothelium *in vivo*^38^, F-actin fibres in endothelial cells were located near the cell–cell interface and aligned with the flow direction as well (Fig. 3e). Further, adherens junctions visualized with VE-cadherin staining confirmed the continuity of cell–cell interface (Fig. 3f). In addition, the microfluidic platform with a well-designed geometry enabled to better control the direction of magnetic force relative to the shear stress and the preformed F-actin fibres.

### Effects of intracellular magnetic force *in vitro*

**Figure 4:**
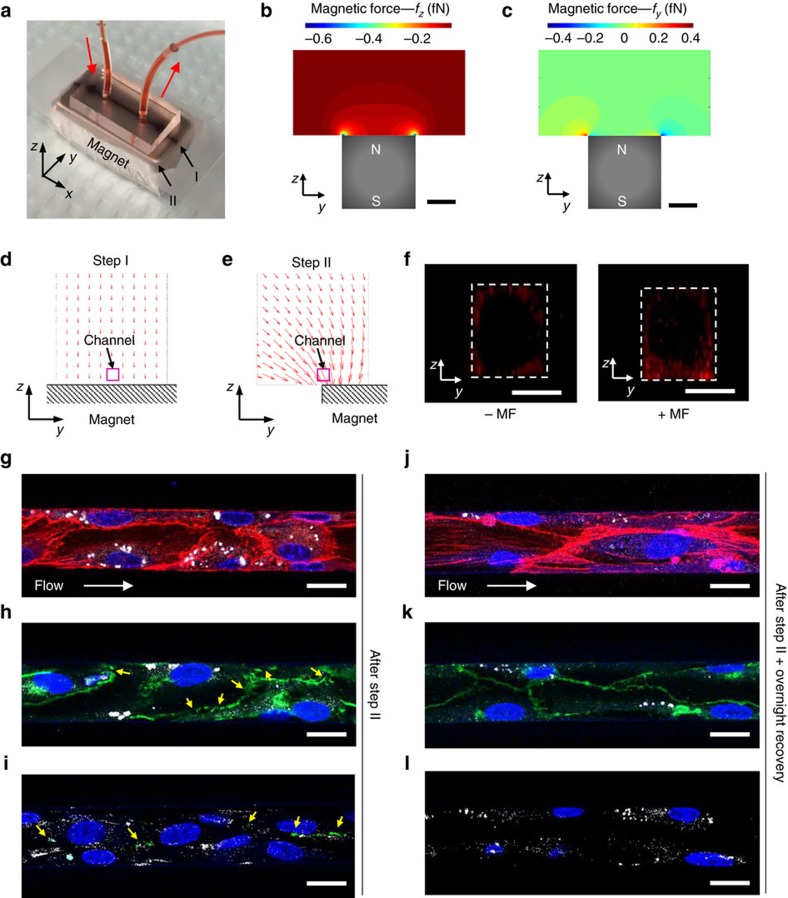
The effect of magnetic force on the endothelium *in vitro*. (**a**) Macroscopic view of a microfluidic device placed on an N52-grade rare-earth magnet (*W* × *H* × *L*=1/2″ × 1/2″ × 1″). The midline of the magnet provides the reference for positioning the microfluidic channels in the magnetic field. The arrows indicate the flow direction. (**b**,**c**) Simulated distribution of magnetic force on MNPs in *z* and *y* directions. At the midline of the magnet, *f*_*z*_ is large and homogeneous, while *f*_*y*_ is negligible. *f*_*y*_ and *f*_*z*_ increase toward the edge of the magnet. Scale bar, 5 mm. (**d**,**e**) The two-step magnetic treatment of the microfluidic channels and the corresponding force distribution. In step I, the microfluidic channel is aligned with the centre line of the magnet for 2 h, while in step II, it is aligned with one edge of the magnet for 1 h. (**f**) Representative confocal images of the cross-section of the endothelium following step I without or with the magnetic field. The magnetic force increased the uptake of MNPs by endothelial cells at the bottom of the channel. Dashed white lines mark the boundary of the channels. Scale bar, 20 μm. In step II, the endothelialized channels were moved to the edge of the magnet for 1 h. After that, the endothelialized channels were either directly fixed and stained for actin and VE-cadherin, or cultured overnight under flow before staining. Immunostaining indicates that the two steps of magnetic force exposure led to disorganized endothelial actin filaments (**g**, red) and disrupted adherens junctions (**h**, green. Yellow arrows indicate the disrupted adherens junctions), while step I alone did not cause any change in actin organization (Supplementary Fig. 8). In addition, the vascular endothelium became permeable to antibody molecules as visualized by collagen I staining (**i**, green). After overnight recovery without magnetic force, the endothelial actin filaments reorganized (**j**, red) and the continuous adherens junctions were restored (**k**, green). No collagen I staining was detectable, indicating the blockade of the endothelium to the antibody (**l**, green). Blue, nuclei; white, MNP. Scale bar, 20 μm.

To determine the effects of magnetic force on the uptake of MNPs and the organization and alignment of preformed actin filaments in endothelial cells under flow conditions, we placed an NdFeB block magnet under the microfluidic device, with its magnetization axis in the vertical direction (*z* axis) and its longitudinal direction (*x* axis) aligned with the flow direction in the microfluidic channel (Fig. 4a–c). Due to the symmetry of magnetic field in the *x* direction, MNPs in the microfluidic channel only experience magnetic forces in the *z* and *y* directions: *f*_*z*_ is perpendicular to both the flow direction and the bottom surface of the channel and *f*_*y*_ is perpendicular to the flow direction but tangential to the bottom surface. When the microfluidic channel is placed along the midline of the magnet (setting I, Fig. 4d), *f*_*z*_ and *f*_*y*_ are nearly uniform throughout the channel (Fig. 4b,c). A numerical simulation indicates that for a 16 nm MNP at this position, *f*_*z*_ is 6.7 × 10^−17^ N, while *f*_*y*_ is negligible (Fig. 4b,c; Supplementary Fig. 7). *f*_*z*_ drives MNPs toward the endothelial cells on the bottom surface of the channel. When the channel is aligned with the edge of the magnet (setting II, see Fig. 4e), the magnitude of *f*_*z*_ increases to 8.6 × 10^−16^ N and *f*_*y*_ becomes 4.1 × 10^−16^ N (Fig. 4b,c; Supplementary Fig. 7). To better elucidate the effects of magnetic force on endothelial cells, these two force settings were used for enhancing MNPs internalization and applying a magnetic force to intracellular MNPs, respectively.

The uptake of MNPs (16 nm/DiI) by endothelial cells under flow (*V*_average_=0.46 mm s^−1^) increased significantly compared with those cultured in static (Supplementary Fig. 8). However, a further tenfold increase in the flow velocity had no significant effect on the uptake rate, suggesting that cell internalization is the rate-limiting step under flow. With the external magnetic forces at setting I, the uptake of MNPs under the flow condition was further increased by a factor of 2.3, and a larger amount of MNPs was taken up by endothelial cells at the bottom of the channel than at other locations (Fig. 4f; Supplementary Fig. 9). However, *f*_*z*_ alone did not cause any detectable change in actin filaments (Supplementary Fig. 10).

In subsequent experiments, after endothelial cells in the microfluidic channel were loaded with MNPs using setting I (Fig. 4d, step I), the channels were incubated with setting II, where MNPs experience forces in both the *y* direction (*f*_*y*_) and *z* direction (*f*_*z*_) that are ∼3–4 orders of magnitude of the gravitational force (Fig. 4e, step II). Here we found that after endothelial cells were exposed to *f*_*y*_ for 1 h, F-actin fibres along the flow direction were reduced (Fig. 4g) as compared to the control condition in which no magnetic force is applied (Fig. 3e). In addition, the distribution of VE-cadherin became discontinuous and diffuse, indicating the disruption of adherens junctions at intercellular interfaces (Fig. 4h). Interestingly, after 12 h incubation with flow upon the withdrawal of magnetic force, endothelial cells reformed F-actin fibres along the flow direction and VE-cadherin returned to its original distribution, that is, continuous and condensed between adjacent cells (Fig. 4j,k). These data suggest that endothelial cells can simultaneously mechanosense the flow-induced shear stress and the intracellular MNP-exerted force, and the relative direction of these two forces modulates F-actin dynamics. Comparison between Figs 3e and 4g,j indicates that the intracellular magnetic force was large enough to induce a reversible change in actin cytoskeleton and the changes in actin filaments led to a temporary disruption to endothelial adherens junctions rather than permanent alterations to endothelial function.

To examine whether the magnetic force-induced disruption of the endothelial adherens junction alters the barrier function of the endothelium, the endothelialized microfluidic channel subjected to the magnetic treatment (step I and step II described above) was infused with an antibody against collagen I immediately after exposure to magnetic field or after the endothelial channel recovered overnight. The microfluidic channel was coated with collagen I to support the growth of endothelial cells and thus located at the basal side of the endothelium. An antibody (∼150 kD) is significantly larger than a serum albumin molecule (66 kD) and therefore cannot diffuse through intact endothelial cell–cell junctions. We found that collagen I was positively stained after the magnetic treatment, indicating that the endothelial layer became permeable to the antibody (Fig. 4i). In contrast, the anti-collagen staining was not detectable after recovery of adherens junction (Fig. 4l). These results demonstrate that the magnetic force-induced disruption of endothelial adherens junctions caused the endothelial layer becoming permeable to large protein molecules.

### *In vivo* magnetic switch of vascular permeability

**Figure 5:**
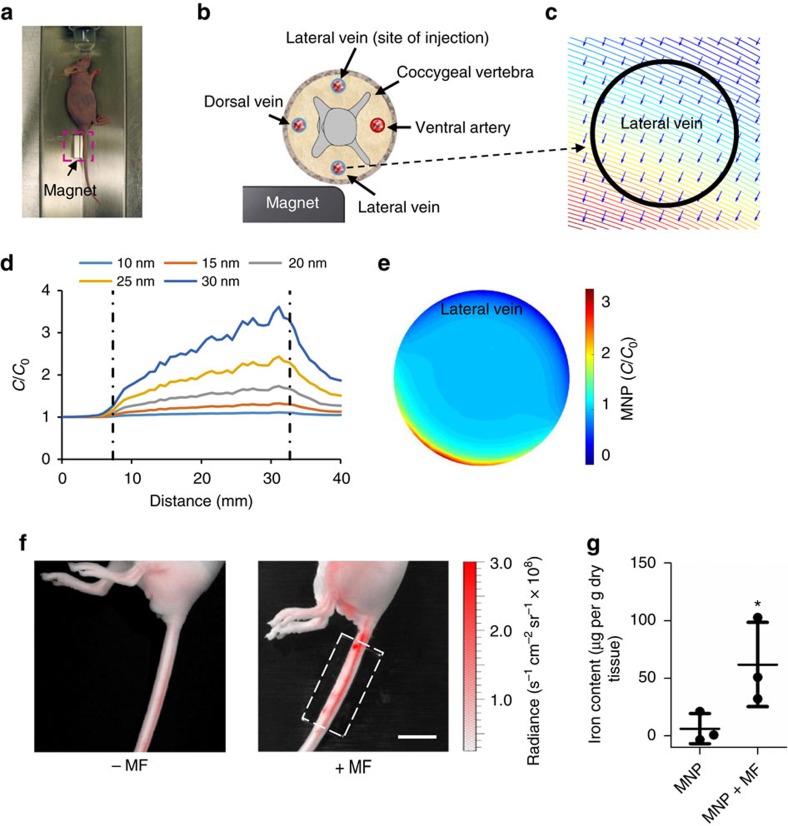
*In vivo* magnetic targeting. (**a**) Set-up of the *in vivo* experiment. An athymic nude mouse was anaesthetized with isoflurane with one of its lateral tail veins aligned with the edge of a rare-earth magnet. (**b**) A schematic diagram of the mouse tail and the magnet. (**c**) Simulated distributions of magnetic flux density and magnetic force. The circle represents the lateral tail vein near the magnet. (**d**) Simulated concentration profiles of MNPs of different sizes on the vessel wall close to the edge of the block magnet. Dashed lines mark the ends of the block magnet. Fluctuations in the concentration are caused by the interpolation of numerical results. (**e**) Simulated concentration profile of 33 nm MNPs in the cross-section of the lateral tail vein. (**f**) Magnetic targeting of 33 nm MNPs labelled with DiR. The dashed box marks the segment of the tail next to the magnet. The NIR fluorescence signal (ex/em=710 nm/780 nm) was pseudocoloured as indicated with the colour bar. Accumulation of MNPs increased along the blood flow direction from the distal to the proximal end of the tail. Scale bar, 10 mm. (**g**) MNPs in the mouse tails quantified by a superconducting quantum interference device (SQUID; mean±s.d.; *n*=3; *P<0.05).

We further tested the concept of magnetic control of vascular permeability *in vivo* in a mouse lateral tail vein model in athymic nude mice. MNPs were injected into the systemic circulation from the distal end of a mouse lateral tail vein, and the opposite lateral tail vein was placed along an NdFeB block magnet (Fig. 5a–c). Our initial studies and computational numerical simulations showed that magnetic targeting in the lateral tail vein required a higher field gradient and larger MNPs due to the increased venous blood velocity and a longer distance from the vessel to the magnet (Fig. 5d,e; Supplementary Figs 12–15). Efficient magnetic targeting was achieved using 33 nm MNPs with a higher magnetic force of ∼5 × 10^−15^ N at the edge of the magnet, as shown by *in vivo* near-infrared fluorescence imaging of MNPs in the mouse tail (Fig. 5f). We further quantified the amount of MNPs in the mouse tail using a superconducting quantum interference device (SQUID), and found that the iron content in the tail (62±37 μg g^−1^ dry tissue) that subjected to applied magnetic field was significantly higher than that without (6.3±13.1 μg g^−1^ dry tissue). Histological examination showed that MNPs accumulated primarily in the large veins and small venules but not in the arterioles, presumably due to the higher arterial blood flow speed. It also indicated that MNPs were only located in the endothelium, but not in the interstitial tissues in the *in vivo* experiments (Supplementary Fig. 16). These results confirm the effective magnetic targeting of MNPs in the mouse tail.

**Figure 6:**
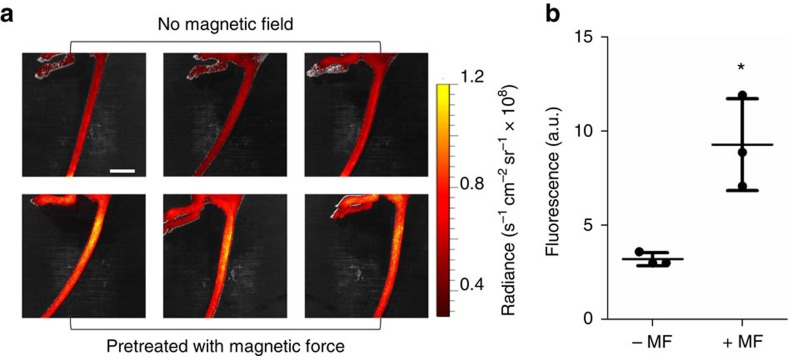
Magnetic enhancement of vascular permeability *in vivo*. Athymic nude mice were injected with 33 nm MNPs through one later tail vein and the one on the opposite site was exposed to the magnetic treatment. After that, the mice were injected with ICG (ex/em=710 nm/810 nm). (**a**) Distribution of ICG without and with the magnetic treatment. The mice subjected to the magnetic treatment showed enhanced ICG accumulation in the area adjacent to the magnet. Scale bar, 10 mm. (**b**) Quantification of ICG accumulation based on fluorescence intensity (mean±s.d.; *n*=3; *P<0.05).

Vascular permeability was evaluated *in vivo* by monitoring the accumulation in mouse tail of circulating indocyanine green (ICG), a small near-infrared fluorophore used in clinical examinations of vessel leakage in ophthalmic angiography^39^. ICG signal is a reliable measure of the altered permeability in vasculature, since ICG molecules in the plasma bind to lipoproteins and protein-bound ICG extravasates from leaky blood vessels but not the normal ones^40^. In this study, after the mice were injected with unlabelled MNPs, the lateral tail vein was placed on the magnet for 2  h. Following exposure to the magnetic field, the mice received a bolus injection of ICG through the tail vein and the distribution of ICG was monitored via *in vivo* imaging. The fluorescence signal of ICG increased in the tail of the mice subjected to the magnetic field (Fig. 6a,b). Enhanced ICG signals correlated well with the location of the magnet but were not confined to the lateral vein, suggesting that extravasation also occurred from surrounding microvessels. In the control mice without the magnetic field, MNPs alone had no effect on ICG distribution. Taken together, these results demonstrate that an external magnetic field can induce local accumulation of MNPs in the target tissue and increase vascular permeability in vessels within the area where a magnetic field is applied, which is likely due to intracellular magnetic force-induced disruption of endothelial adherens junctions.

## Discussion

Overcoming the endothelial barrier is paramount to the treatment of many diseases. In particular, it is essential to the development of new biological therapies that involve engineered proteins, nucleic acids as well as multifunctional nanoparticles as drug carriers. To date, the main rationale for delivering these large pharmacological agents to the target tissue or cells is the enhanced permeability and retention effects pertaining to tumour tissue at an active growing phase. Otherwise, for large drug molecules and drug carriers, their systemic delivery is limited to cells in the circulatory system. Here we developed a new strategy in which biocompatible MNPs combined with a well-designed magnetic field can provide an externally controllable mechanical cue to activate the paracellular transport pathway of vascular endothelium at the desired location^20^. The applied magnetic force on endothelial cells is determined by the amount of intracellular MNPs, their size and the applied magnetic field. Previous studies have shown that a focused magnetic field gradient can be generated at any location in the body^41^,^42^; whether these gradients are sufficient for magnetic capture or to generate the forces described here will depend on the properties of the nanoparticles, the flow rate in the vessels and the gradient generated at that point. In contrast to ultrasound-based methods, magnetic fields are not attenuated by biological tissues, enabling better control of targeted delivery into deep tissue. In addition, the pharmacokinetics and interactions of MNPs with the vascular endothelium can be optimized by fine-tuning nanocrystal synthesis and surface modification, owing to a large repertoire of nanofabrication techniques.

There are two major aspects concerning the potential clinical application of the method developed in this study. The first is to deliver MNPs to the targeted endothelium, of which magnetic targeting is just one of the many ways. Other approaches include conjugating targeting ligands or cell-penetrating peptides to MNPs and direct injection (when permitted). The second is to apply magnetic force to intracellular MNPs *in vivo* (in deep tissue), possibly using a clinical-grade magnetic device in which multiple magnets of different strengths can be placed on the patient at specific anatomic sites to yield the optimal magnetic field strength and distribution to enhance delivery at the desired location^43^,^44^. Magnetic enhancement of vascular permeability thus has the potential to be applied to any tissue or organ of interest, regardless of the pathophysiological state.

Endothelial mechanotransduction of the blood flow-induced shear stress plays a critical role in modulating vascular homeostasis. Dysregulation of the mechanotransduction pathways can trigger pathogenesis of cardiovascular diseases, especially atherosclerosis^45^. The effects of the shear stress include modulation of the cytoskeleton, intercellular junctions and vascular permeability^46^,^47^. In this scenario, the shear force is exerted on the membrane of endothelial cells, and the mechanosensing has been hypothesized to be initiated through cell surface mechanotransducers, such as carbohydrate-rich glycocalyx and mechanosensory complex comprising of platelet endothelial cell adhesion molecule 1, VE-cadherin and VEGF receptor 2 (refs 48, 49). However, MNP-generated intracellular forces are more ‘body-force' like rather than surface forces, and the mechanisms underlying magnetic force-induced mechanosensing and mechanotransduction remain elusive. Our study demonstrates that the force applied through intracellular MNPs can trigger the reorganization of F-actin fibres and disrupt endothelial adherens junctions. The phenotypic changes induced by the transient application of intracellular forces are found to be similar to those induced by a rapid change in the shear stress. Microscopic examination shows that the intracellular MNPs accumulated in the lysosomes, which have certain association with actin filaments^50^. Our results suggest that the MNP-induced intracellular forces may serve as a new way to apply distributed forces inside the cell, which can be transmitted directly to the cytoskeleton. Therefore, MNPs combined with an externally controllable magnetic field provide a novel technique to trigger mechanoresponses of cells and can potentially offer new insights into mechanobiology.

Conventional drug delivery methods employ polymers or nanoparticles as drug carriers, which are limited to therapeutic agents with specific properties for conjugation, adsorption or encapsulation. In contrast, our approach provides a means to remotely alter the cytoskeletal organization of endothelial cells via intracellular magnetic forces, thus enhancing focal vascular permeability and opening the gate for extravasation of all circulating agents. It, therefore, extends targeted delivery to the vast majority of injectable therapeutic agents. It should also be noted that disrupting endothelial cell–cell junctions may expose cell surface proteins and basement membrane that are otherwise inaccessible to the blood circulation, potentiating additional molecular markers for targeted delivery.

In conclusion, we demonstrate that the permeability of vascular endothelium can be increased using an external magnetic field to temporarily disrupt endothelial adherens junctions through internalized iron oxide nanoparticles, thus activate the paracellular transport pathway and facilitate the local extravasation of circulating substances. The magnetic control method developed in this work has the potential to shift the paradigm of targeted drug delivery by delivering therapeutic agents to specific anatomic regions within the body for a broad range of disease interventions.

## Methods

The investigators were not blinded to allocation during experiments and outcome assessment.

### Materials

Iron(III) acetylacetonate (99.9%), ICG and fibronectin from human plasma were purchased from Sigma-Aldrich. 1,2-Distearoyl-sn-glycero-3-phosphoethanolamine-*N*-[methoxy (polyethylene glycol)-2000] (ammonium salt) (phospholipid–PEG) was purchased from Avanti Polar Lipids. 1,1′-dioctadecyl-3,3,3′,3′-tetramethylindocarbocyanine perchlorate (DiI) and 1,1′-dioctadecyl-3,3,3′,3′-tetramethylindotricarbocyanine iodide (DiR) were purchased from Thermo Fisher Scientific. HUVECs from Lonza were cultured with EGM-2 medium purchased from Lonza. These cells were tested for mycoplasma contamination and authenticated by the vendor.

### Synthesis of magneto-fluorescence nanoparticles

Magnetite nanocrystals were synthesized by thermo-decomposition of iron acetylacetonate^30^,^31^. Transmission electron microscopy (TEM) images of nanocrystals were recorded with a Hitachi H-7500 Transmission Electron Microscope connected to a charge-coupled device camera. The negative staining and TEM procedures were conducted by Robert P. Apkarian Integrated Electron Microscopy Core at Emory University. The size of the nanocrystals was measured as the average size of >200 particles with ImagePro Plus in TEM images. The magnetization of the nanocrystals was measured with a SQUID (MPMS-XL, Quantum Design) at the Advanced Materials Research Institute in the University of New Orleans. Water-dispersible MNPs were generated by coating the nanocrystals with amphiphilic phospholipid–PEG copolymers using a dual solvent exchange method^32^. MNPs were labelled with fluorophores by mixing MNPs and DiI or DiR in deionized water at a weight ratio of 40:1 and incubating at ambient temperature for 12 h. Unbound fluorophores were removed by passing the solution through 0.2 μm syringe filters with HT Tuffryn membrane. The size distribution and the zeta potential of the MNPs were measured with the Möbiuζ Zeta Potential and DLS Detector (Wyatt).

To demonstrate the fluorescent signal is proportional to the iron centration, we measured the fluorescent signals of iron oxide solution at different concentrations with a fluorometer (Supplementary Fig. 1a). To further confirm the fluorescent dye tightly binds to MNPs as a reliable marker, we first incubated fluorescent dye-labelled MNPs with bovine serum albumin for 24 h, and analysed whether the fluorescent signals transferred to BSA using size exclusion chromatography (SEC) analysis (Supplementary Fig. 1b). We then tested whether the fluorescent tags would detach from MNPs and transfer to cells when MNPs are in close contact with the cell membrane due to magnetic force. This was performed at 4 ^o^C to completely inhibit cellular endocytosis and only allow the magnetic force to mediate contact between MNPs and cell membrane (Supplementary Fig. 2).

### Design of magnetic force fields

In this study, all magnetic fields were created using NdFeB rare-earth magnets. Note that the magnetic force exerted on MNPs is determined by the strength and gradient of the magnetic field (Supplementary Methods). As shown in Table 1, different assemblies of magnets were used to generate force fields that could cover the cells in the desired range. In the cellular uptake study, the cells were cultured in a chamber glass placed on top of an array of cylindrical magnets covering the entire chamber. In all other studies, one or two block magnets were used.

### Effects of magnetic force on static cell culture *in vitro*

Lab-Tek two-well chambered coverglass was coated with rat tail collagen type I solution at 0.1 mg ml^−1^. HUVECs were seeded onto the coverglass at a density of 1 × 10^5^ cells per well. After 24 h, the cells were incubated with MNPs at a concentration of 50 μg of Fe per ml for 2 h. For magnetically enhanced cellular uptake, the coverglass was placed on an array of NdFeB cylindrical magnets. After incubation, the cells were washed with PBS and detached by treatment with trypsin-EDTA. The fluorescence intensity of intracellular MNPs was quantified by flow cytometry. To measure the iron content of MNPs in the cells, the cells were counted and centrifuged. After the cell pellets were dried under vacuum, the cells were digested with 12 M hydrochloric acid at 37 °C for 1 h. The iron content was quantified using a ferrozine assay. The iron content in each MNP was calculated using the size of the nanocrystals determined in TEM with the assumption that the nanocrystals consisted of pure magnetite^51^.

To study the effects of intracellular magnetic force on the cytoskeleton, the cells were loaded with MNPs, detached and seeded in a new chamber glass. After the cells were cultured overnight, the chamber was incubated on an NdFeB block magnet for 2 h. At the end of experiments, the cells were fixed with 4% paraformaldehyde in PBS and the actin fibres were stained with phalloidin-alexa fluor 488 (1:20; Life Technologies).

### Fabrication of endothelialized microfluidic channels

To fabricate the microfluidic devices, a top polydimethylsiloxane (PDMS) piece with microfluidic channel features was fabricated using standard photolithography and soft lithography. After activated with oxygen plasma, the PDMS layer was covalently bonded onto a piece of coverglass. The microfluidic channels were first coated with a rat tail collagen type I solution at 1 mg ml^−1^ for 1 h, followed by coating with human fibronectin solution at 20 μg ml^−1^ for another 1 h. HUVECs (passage 5–8) were seeded into the microfluidic channels at a concentration of ∼5 × 10^6^ cells per ml. After incubated at 37 °C for 3 h, the microfluidic device was connected to a flow pump filled with cell culture medium (EGM-2, Lonza). The wall shear stress in the smallest channels was set to designated values by adjusting the flow rate. The endothelial cells were cultured under flow for 2–3 days before experiments.

### Effects of magnetic force in microfluidic channels

MNPs were diluted to 50 μg of Fe per ml using EGM-2 medium and infused through the endothelialized microfluidic channels with/without a magnet placed underneath the channels (the detailed simulation of the magnetic field and magnetic force in the channel were provided in the Supplementary Materials). To elucidate the effect of flow on MNP uptake by endothelial cells, MNPs were infused into the endothelialized microfluidic channels with a shear stress varying from 0 to 10 dyn cm^−2^ for 2 h. At the end of experiments, 4% paraformaldehyde in PBS was infused through the channels to fix the cells. The *z*-stack confocal images of endocytosed MNPs were taken, and the fluorescence intensity was quantified using Image J on the three-dimensional rendering images. To study the role of magnetic forces in MNP uptake by endothelial cells and endothelial barrier function, MNPs were first infused into the endothelialized microfluidic channels with the channels placed along the midline of the magnet (setting I, Fig. 4d) with a shear stress of ∼6.5 dyn cm^−2^ for 2 h. After that, the endothelialized microfluidic channels were aligned with the edge of the magnet and infused with cell culture medium only for 1 h (setting II, Fig. 4e). To study whether the disruption of adherens junctions is reversible, the endothelialized microfluidic channels were further cultured with a shear stress of ∼6.5 dyn cm^−2^ for 12 h. To stain VE-cadherin, the endothelialized channels were fixed with 4% paraformaldehyde, washed with PBS, and blocked in PBS containing 1% BSA, followed by perfusing with mouse anti-human VE-cadherin (1:100, Santa Cruz, cat#: sc-9989) for 2 h. Then, the channels were incubated with antibody at 4 °C for 12 h. After flushed with PBS containing 1% BSA, the channels were perfused with rabbit anti-mouse Alexa Fluor 488 (1:100, Life Technologies, cat#: A11001) in 1% BSA for 2 h. After further washes with PBS, the channels were perfused with hoescht 33342 (1:1,000; Life Technologies) and phalloidin-AF 647 (1:20; Life Technologies) for 1 h. Collagen I in the microfluidic channels were stained with a monoclonal mouse anti-collagen I antibody (Abcam, cat#: ab90395) using the same procedure.

### *In vivo* permeability study

All animal procedures were approved by the Institutional Animal Care and Use Committee at Rice University. Athymic nude mice (Crl: NU(NCr)-Foxn1^nu^) were purchased from Charles River Laboratories. Age-matched (4–5 weeks) female animals were randomly assigned to experimental groups throughout all experiments. In the magnetic targeting experiment, 33 nm MNPs labelled with DiR were diluted to 1 mg of Fe per ml^−1^ with water. The osmotic pressure of the solution was adjusted with dextrose. The mice were anaesthetized with isoflurane. A measure of 0.15 ml of MNPs was injected through the distal end of a lateral tail vein. The mouse tail was taped onto the magnets to align the contralateral tail vein with the edge of the magnet (Fig. 5a). After 2 h, fluorescence images of live mice were acquired with IVIS spectrum imaging station (ex/em=710 nm/780 nm). To quantify MNPs accumulated in the tail, the mice were killed and infused with 10 ml of PBS to remove circulating MNPs. Then, the tails were dissected and cut into 10 mm segments and dried under vacuum overnight. The magnetization of the tissue was measured at 10,000 Oe with SQUID (MPMS, Quantum Design). Untreated mouse tails were used as the reference. The iron content of MNPs was calculated by dividing the net magnetization of the tissue by the magnetization of MNPs.

In the vascular permeability study, 0.15 ml of unlabelled 33 nm MNPs was injected through the distal end of a lateral tail vein. The contralateral tail vein was treated with magnets for 2 h. After that, 0.05 ml of ICG (2 mg ml^−1^ in water with dextrose) was injected. Fluorescence images of live mice were acquired with IVIS spectrum imaging station at 1 h after ICG injection (ex/em=710 nm/810 nm).

### Statistics

All results are expressed as the mean±s.d. Biological replicates were used in all experiments. Data were analysed using the Student's *t*-tests or one-way analysis of varience with Dunnett's *post hoc* test. The threshold for statistical significance was *P*<0.05. GraphPad Prism (GraphPad Software) was used for all the calculations. No statistical methods were used to pre-determine the sample size of the experiments.

### Data availability

The data that support the findings of this study are available from the corresponding authors upon reasonable request.

## Additional information

**How to cite this article:** Qiu, Y. *et al*. Magnetic forces enable controlled drug delivery by disrupting endothelial cell-cell junctions. *Nat. Commun.* **8**, 15594 doi: 10.1038/ncomms15594 (2017).

**Publisher's note:** Springer Nature remains neutral with regard to jurisdictional claims in published maps and institutional affiliations.

## Supplementary Material

Supplementary Information Supplementary Figures, Supplementary Tables, Supplementary Methods and Supplementary References.

## Figures and Tables

**Table 1:** The properties of magnets used in *in vitro* and *in vivo* experiments.

**Experiment**	**Dimension of magnet**	**Residue induction (T)**
*In vitro* *static cell culture*		
Cellular uptake	Cylinder (*d* × *l*=1/10″ × 1/4″)	1.32
Magnetic pulling	Block (*l* × *w* × *h*=1″ × 1/4″ × 1/4″)	1.48
Microfluidic channels	Block (*l* × *w* × *h*=1″ × 1/2″ × 1/2″)	1.48
*In vivo* lateral tail vein	Block (*l* × *w* × *h*=1″ × 1/4″ × 1/4″)	1.48
