# Supplementary material for: Magnetic forces enable controlled drug delivery by disrupting endothelial cell-cell junctions
Source: Nat Commun. 2017 Jun 8;8:15594. doi: 10.1038/ncomms15594 (PMC5472756; doi:10.1038/ncomms15594)
Supplement: Supplementary Information — Supplementary Figures, Supplementary Tables, Supplementary Methods and Supplementary References. [file ncomms15594-s1.pdf]

Supplementary Methods

Modeling of magnetic field

In this study, the magnetic field was generated with N52 grade nickel-plated neodymium block magnets (K&J Magnetics). The residual flux density of the magnets is 1.48T. Magnets with two different sizes, W×H×L = 1/2″×1/2″×1″ or 1/4″×1/4″×1″, were used in *in vitro* and *in vivo* experiments respectively. We assumed that all magnets were uniformly magnetized along one of the short axes and neglected the nickel coating (15-21 μm) and shape defects in computation. The magnetic field was calculated using the linear superposition principle. For a point dipole $\boldsymbol{m}$, its magnetic field is

$$\boldsymbol{B}(\boldsymbol{r}) = \frac{\mu_0}{4\pi}\left(\frac{3\boldsymbol{r}(\boldsymbol{m}\cdot\boldsymbol{r})}{r^5} - \frac{\boldsymbol{m}}{r^3}\right) \quad (1)$$

where $\boldsymbol{B}(\boldsymbol{r})$ is the magnetic flux density, μ_0 is the vacuum permeability, $\boldsymbol{r}$ is the vector from the dipole to the point, and r is the distance. The magnetic field of a block magnet can be calculated by integrating above equation over the whole magnet,

$$\boldsymbol{B}(\boldsymbol{r}) = \frac{1}{4\pi}\iiint\left(\frac{3\boldsymbol{r}(\boldsymbol{B_{res}}\cdot\boldsymbol{r})}{r^5} - \frac{\boldsymbol{B_{res}}}{r^3}\right) dV \quad (2)$$

where $\boldsymbol{B}_{res}$ is the residual magnetic flux density. The magnetic flux density and its gradient were calculated numerically using a Matlab program developed in-house. To avoid singularity in numerical integration, the computed domain started from 0.2 mm above the magnet, which is the thickness of the glass coverslips used in the microfluidic device. The magnetic flux density and the field gradient used *in vitro* and *in vivo* are shown in **Supplementary Figures 4** and **5**. The magnetic flux density is similar on the surface of the magnets, while the maximum field gradient occurs at the sharp edges.

Modeling of magnetic force on MNPs

To calculate the magnetic force on MNPs, each MNP is approximated as an equivalent point dipole **m**,

$$\boldsymbol{m} = V_{\boldsymbol{p}}\boldsymbol{m_p} \quad (3)$$

where V_p and $\boldsymbol{m}_p$ are the volume and the magnetization of MNP respectively. For the MNPs used in this study, the magnitude of $\mathbf{m}_p$ is determined by local magnetic field strength as shown in Figure **1B** and the direction is aligned with $\boldsymbol{B}$. The magnetic force on each MNP is [1, 2],

$$\boldsymbol{F_m} = \boldsymbol{\nabla}(\boldsymbol{m}\cdot\boldsymbol{B}) \quad (4)$$

Supplementary Figures 7 and **12** show the magnetic force field calculated for 16 nm MNPs in the magnetic field used *in vitro* and for 33 nm MNPs in the magnetic field *in vivo* respectively. In line with the field gradient maps

shown in **Supplementary Figures 4** and **5**, MNPs will experience the largest magnetic force at the sharp edges of the magnets.

Two configurations of the magnetic field were used in *in vitro* experiments to demonstrate the effects of different forces since the microfluidic channels are rectangular. At the center of the magnet, the magnetic force was only in the z direction, perpendicular to the bottom of the channel. The increase in the MNP uptake by the cells at the bottom was apparent compared with the other three surfaces. On the other hand, the bottom cells experienced a large tangential force when the channels were moved to the edge of the magnet, which contributed to the magnetic disruption of actin filaments.

Modeling of transport of MNPs in the mouse lateral tail vein with an external magnetic field

Transport of MNPs in solution was modeled with a diffusion equation with the Brownian motion of MNPs defined by the Stokes-Einstein equation,

$$D = \frac{k_B T}{6\pi\eta r_H} \tag{5}$$

where D is the diffusion coefficient of MNPs, k_B is the Boltzmann's constant, T is the absolute temperature, η is the viscosity of the medium, and r_H is the hydrodynamic radius of MNPs. In this study, r_H of MNPs was measured using dynamic light scattering. Body forces were modeled by introducing drift velocities in the diffusion equation[2].

Our model included the magnetic force generated by rare earth magnets ($\boldsymbol{F_m}$), the viscous force exerted by blood flow ($\boldsymbol{F_f}$) and gravity ($\boldsymbol{F_g}$). The interactions among MNPs and between MNPs and blood cells were neglected. We also assumed the movement of MNPs would not perturb the flow field, nor will it affected the magnetic permeability of the media. The modeling of MNP transport with the flow can thus be solved in three decoupled steps: 1) computation of the magnetic field of rare-earth magnets, 2) computation of the magnetic force field and 3) simulation of the transport of MNPs in flow in the presence of a magnetic force field. The transport of MNPs could be modeled with the following equation,

$$\frac{\partial C}{\partial t} = \nabla \cdot (D\nabla C - \boldsymbol{U}C) \tag{6}$$

where C is the volume concentration of MNP and $\boldsymbol{U}$ is the drifting velocity. Assuming the particle inertia is negligible, we obtain,

$$\boldsymbol{F_m} + \boldsymbol{F_f} + \boldsymbol{F_g} = 0 \tag{7}$$

$\boldsymbol{F_m}$ was calculated as aforementioned.

$$\boldsymbol{F_f} = 6\pi\eta\, r_H\, (\boldsymbol{V_f} - \boldsymbol{U}) \tag{8}$$

where $\boldsymbol{V_f}$ is the velocity of the blood. $\boldsymbol{V_f}$ is calculated as the unidirectional flow in a round tube (the lateral tail vein) with given flow rate and geometry. As mentioned in the main text, the endocytosis of MNPs by the

endothelium is slower than the transport of MNPs in the microfluidic channel. For simplicity, the vessel endothelium was assumed to be an impermeable barrier without taking into account of the uptake of MNPs.

Supplementary Figure 12 shows the magnetic force field calculated for 33 nm MNPs in *in vivo* study. Model geometry, simulated flow profile and magnetic drift velocity of 33 nm MNPs in the lateral tail vein are shown in **Supplementary Figure 13**. The mean inflow velocity was assumed to be 10 mm s^{-1}, which is typical of the blood flow in a vein. The concentration profiles of MNPs in the axial and sagittal cross sections of the vein are shown in **Supplementary Figure 14**. Our simulation showed that the distribution of MNPs rapidly reached equilibrium in a few seconds. MNPs accumulate at the bottom surface of the tube. A highly concentrated region appears near the end of the magnet and the peak concentration is 3.5 folds of the concentration at the entrance.

The results of numerical simulations are summarized in **Supplementary Table 2**.

Supplementary figures

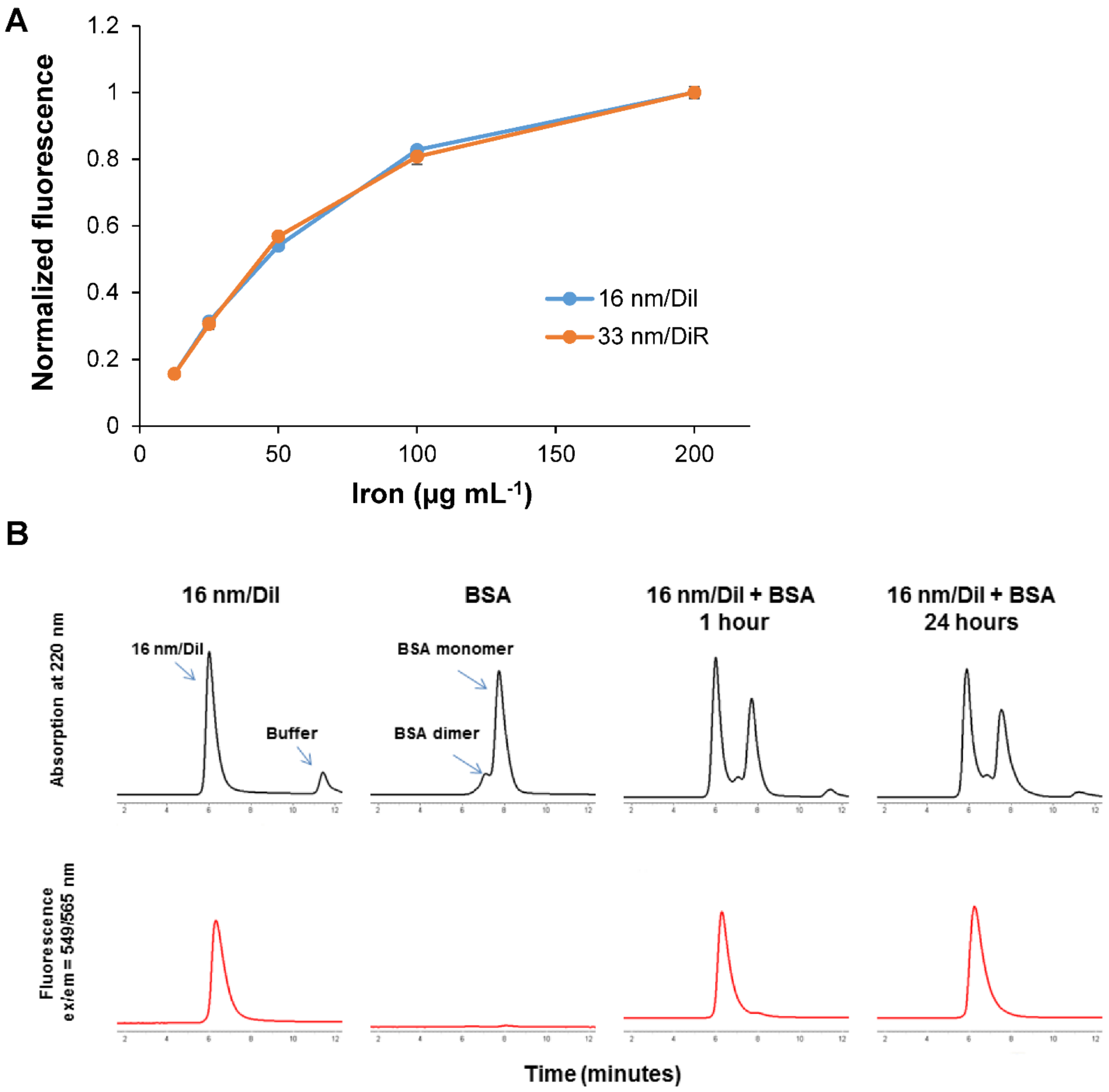

Supplementary Figure 1. Characterization of magneto-fluorescence nanoparticles. A) Calibration curves of fluorescence signals. **B**) Stability of the magneto-fluorescence nanoparticles. MNPs (16 nm/DiI) was incubated with PBS containing 1% BSA at 37°C. The distribution of DiI among the nanoparticles and BSA was analyzed by size exclusion chromatography. Top, light absorption at 220 nm from the nanoparticles and BSA; and bottom, the fluorescence signal from DiI. Our study showed that DiI remained with the nanoparticles after 24-hour incubation with BSA.

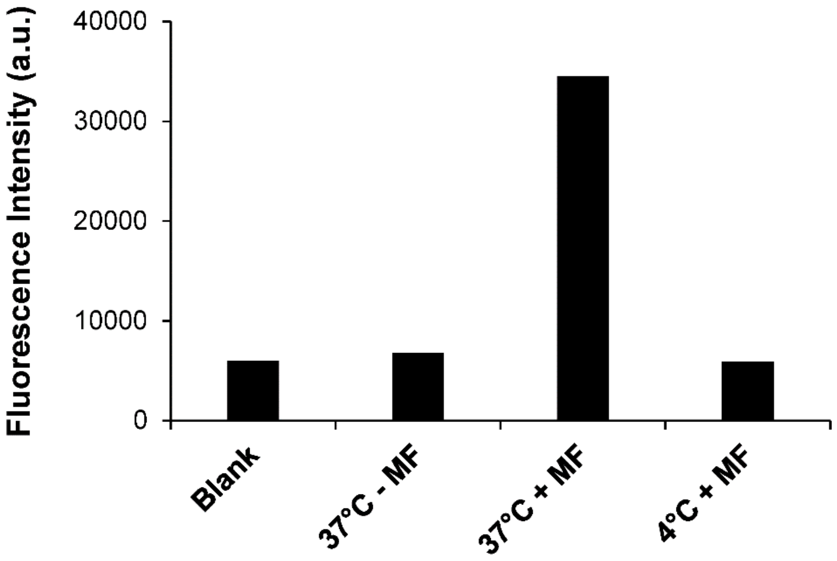

Supplementary Figure 2. **The effect of membrane activity on cellular uptake of MNPs.** The endothelial cells cultured in chambered coverglass were incubated with the medium containing MNPs (16 nm/DiI) under a magnetic field. After two hour incubation, the cells were detached by trypsin-EDTA and the amount of MNPs inside the cells were evaluated by flow cytometry. The magnetic field dramatically increased the uptake of MNPs by the endothelial cells. However, incubation at 4°C, at which there is no cell membrane activity, completely abolished cellular uptake. This indicates that the uptake of MNPs depends on spontaneous membrane invagination. The magnetic force is not sufficient to drive MNPs break through the cell membrane. Also, there was no leakage of DiI from MNPs.

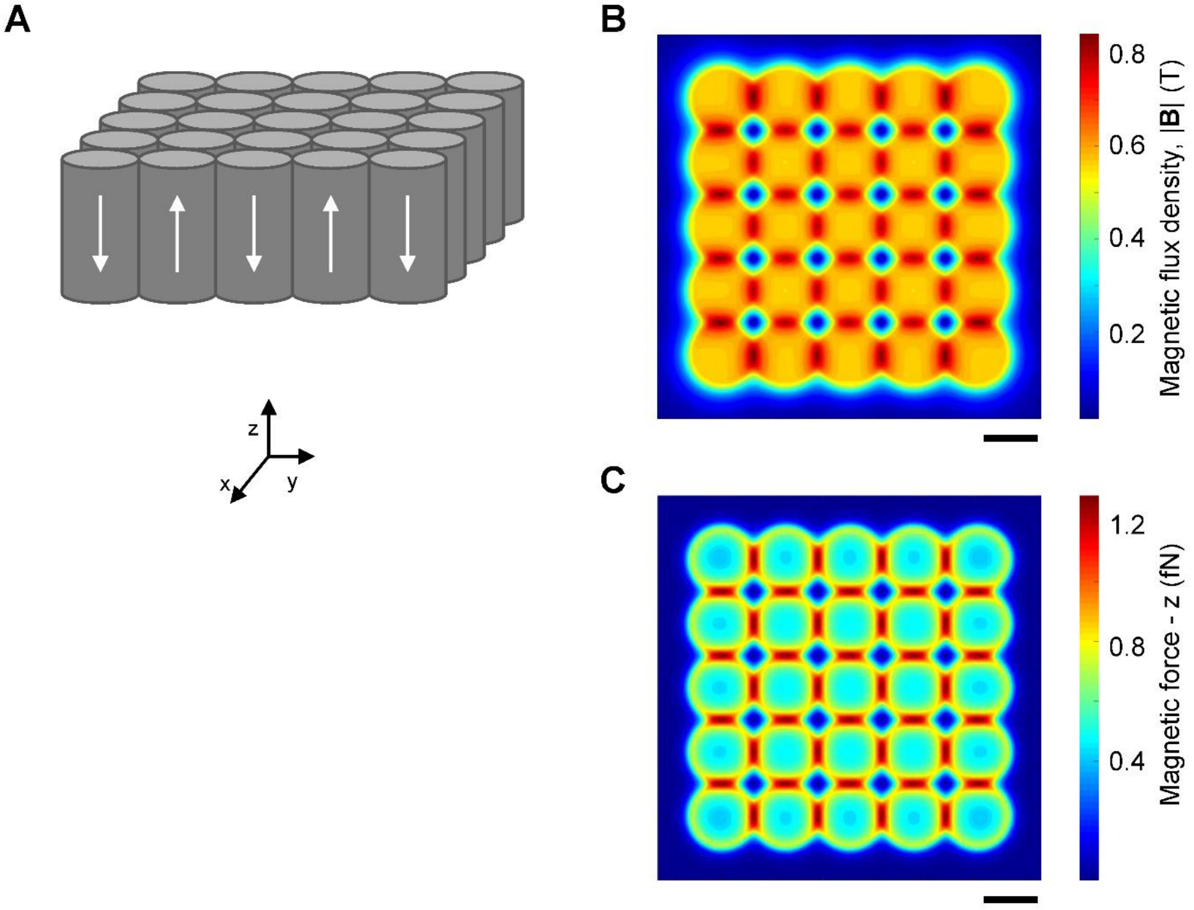

Supplementary Figure 3. The magnetic field for *in vitro* MNP uptake in static cell culture. The cells were cultured in two-well chambered cover glass (1″×1″ per well) placed on a magnetic sheet that provided pulling force for the whole chamber. The sheet was assembled with small cylindrical magnets (H×D = 1/4″×1/10″). For simplicity, only a 5×5 matrix is shown here. **A**) A schematic diagram of the matrix of the magnets. The arrows denote the direction of magnetization. Neighboring magnets had opposing magnetization. **B**) The magnetic flux density. **C**) The magnetic force in the z-direction. Scale bar = 2mm.

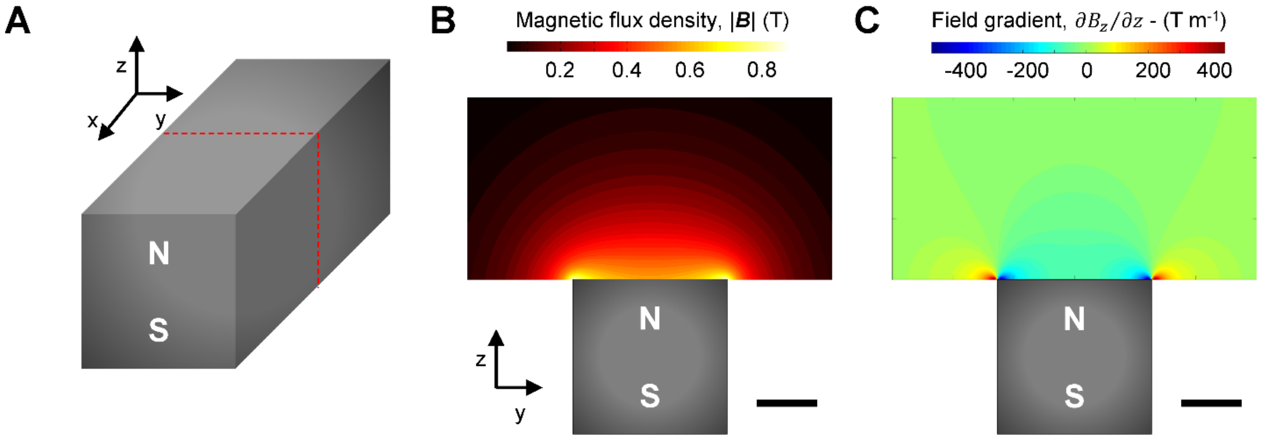

Supplementary Figure 4. The magnetic flux density and the field gradient *in vitro*. **A**) Diagram of the block magnet (W×H×L = 1/2″×1/2″×1″). The magnet is magnetized along the Z direction. **B**) The magnetic flux density. **C**) One component of the magnetic flux density gradient, $\partial B_z/\partial z$. The magnetic flux density and the field gradient within the plane marked with dashed lines in **A**) are plotted. Scale bar = 5 mm.

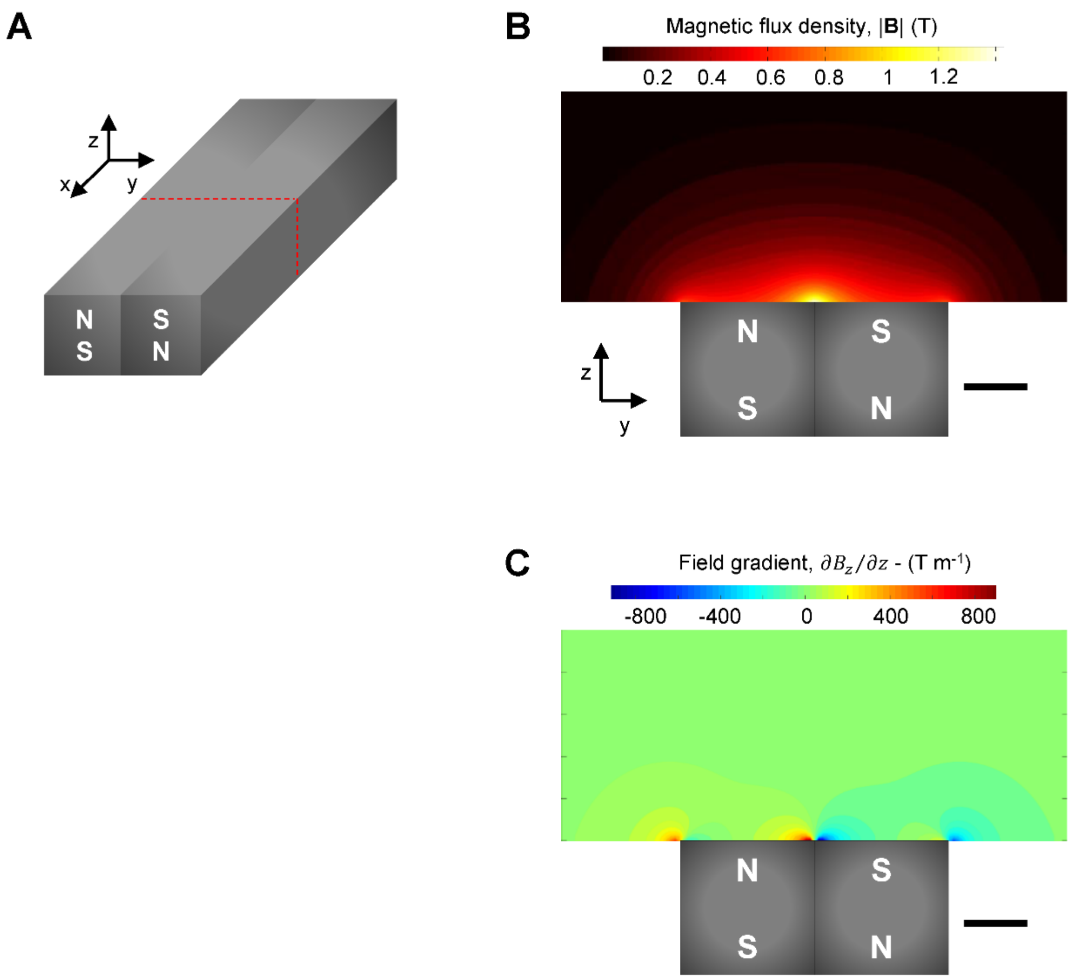

Supplementary Figure 5. **The magnetic flux density and the field gradient *in vivo*.** **A**) Diagram of the magnets (W×H×L = 1/4″×1/4″×1″). A pair of magnets were used in the study. Both magnets are magnetized along the Z direction. **B**) The magnetic flux density. **C**) One component of the magnetic flux density gradient, $\partial B_z/\partial z$. The magnetic flux density and the field gradient within the plane marked with dashed lines in **A**) are plotted. Scale bar = 3 mm.

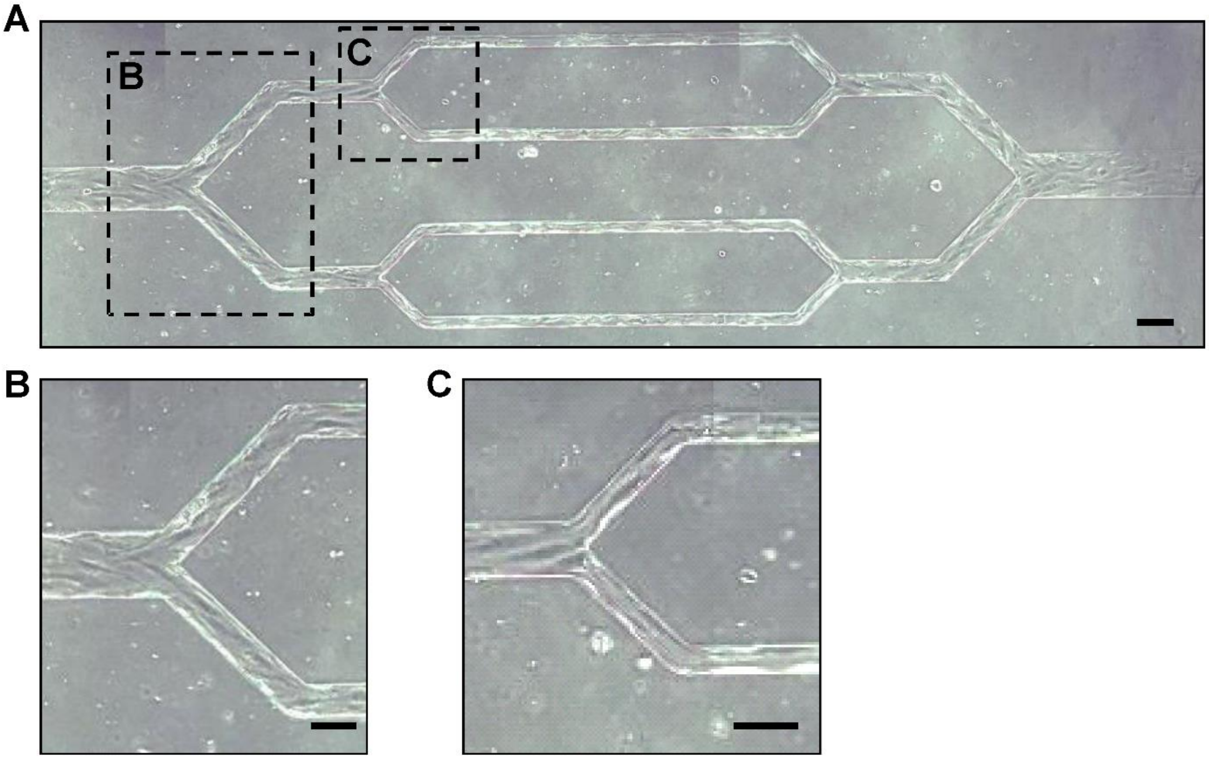

Supplementary Figure 6. Endothelialized microfluidic channels. HUVECs formed a confluent monolayer that covering the inner surface of microfluidic channels after 2-day culture under flow. **A**) A representative bright field image of a full view of endothelialized microfluidic channels. **B** and **C**) Images of box B and C in A with higher magnification, which shows that endothelial cells in the channels aligned with the flow. Scale bar = 50 µm.

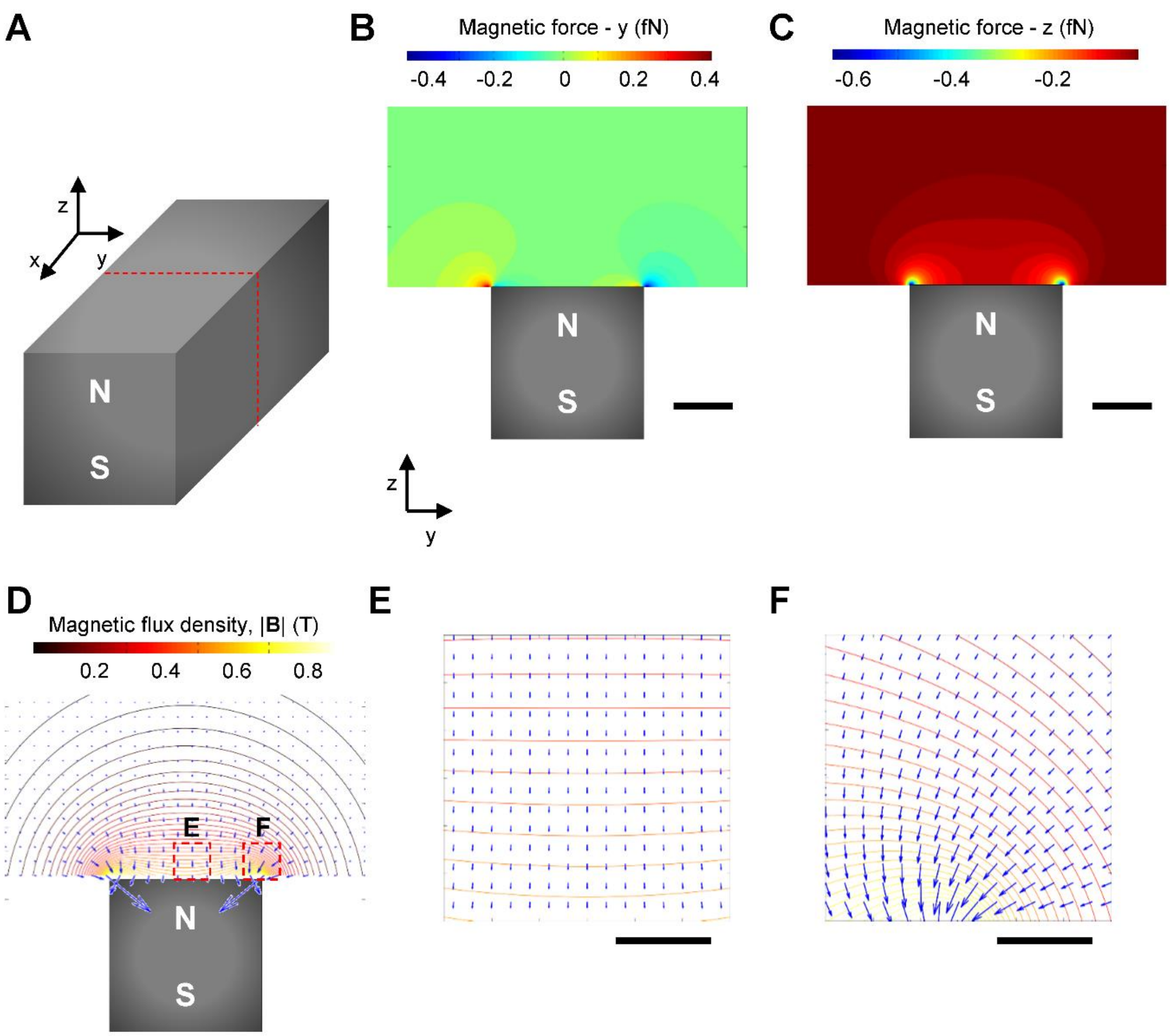

Supplementary Figure 7. The magnetic force field *in vitro*. A) Diagram of the block magnet (W×H×L = 1/2″×1/2″×1″). B) Contour map of magnetic force in the y direction. C) Contour map of magnetic force in the z direction. Scale bar = 5mm. D) Overlaid contour lines of magnetic flux density and magnetic force vectors. Dashed squares mark the two regions used for E) magnetic targeting and F) application of tangential intracellular magnetic force respectively. The force components and vectors within the plane marked with dashed lines in A) are plotted. Scale bar = 1 mm.

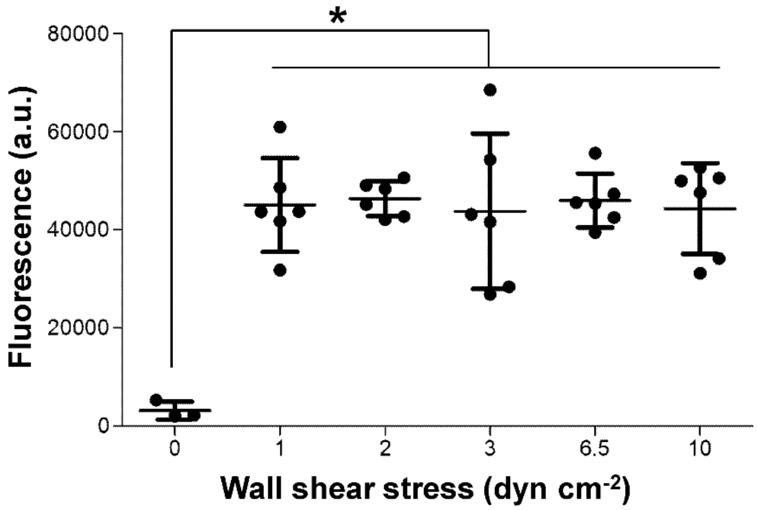

Supplementary Figure 8. Cellular uptake of MNPs in endothelialized microfluidic channels. Endothelial cells in the microfluidic channels internalize more MNPs compared to the static condition, while the magnitude of wall shear stress exhibits no effect on the internalization of MNPs. Data represent mean ± s.d.; n = 3 or 6. Statistical significance was calculated via one-way ANOVA with a Dunnett's post-hoc test. *$p < 0.05$.

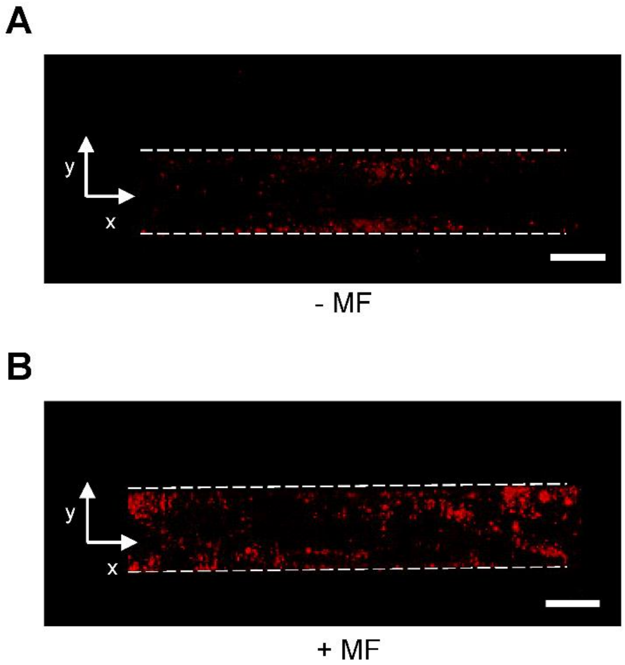

Supplementary Figure 9. Representative 3d-rendering of internalized MNPs by endothelial cells cultured in the microfluidic channels. A) without exposure to external magnetic force and B) after exposed to external magnetic force for 2 hours. Red: MNP. Scale bar = 20 μm.

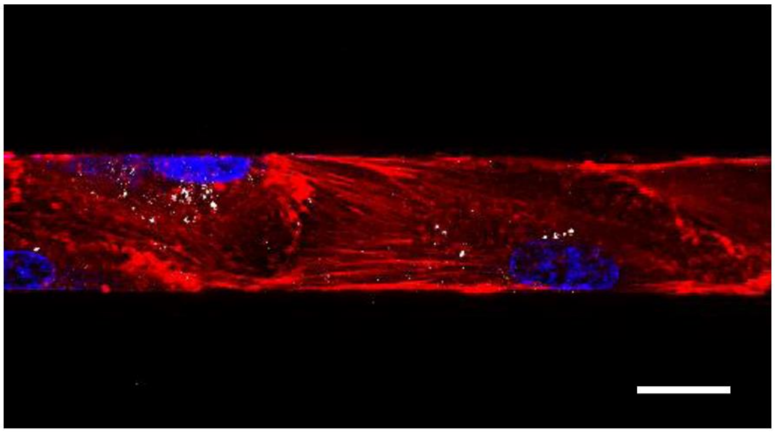

Supplementary Figure 10. Effect of the magnetic force perpendicular to the cell surface. Application of an intracellular magnetic force perpendicular to the cell surface alone for 2 hours did not change the distribution of actin filaments. Red: actin filaments, white: MNP, and blue: nuclei. Scale bar = 20 μm.

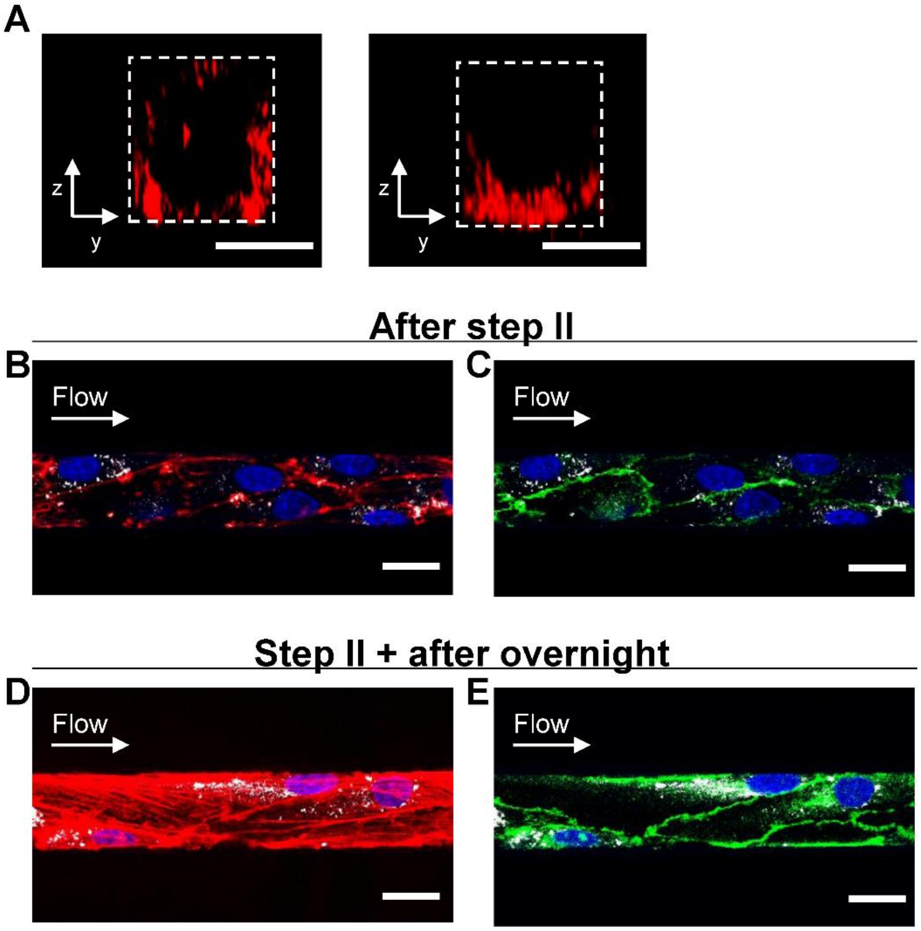

Supplementary Figure 11. Effects of 33 nm MNPs on actin filaments and adherens junctions. (**A**) Representative confocal images of the cross-section of the endothelium following step I (top, without and bottom, with the magnetic field.). The magnetic force increased the uptake of MNPs by endothelial cells at the bottom of the channel. Dashed white lines mark the boundary of the channels. Red: MNP. In Step II, the endothelialized channels were moved to the edge of the magnet for 1 hour. After that, the endothelialized channels were either directly fixed and stained for actin and VE-cadherin, or cultured overnight under flow before staining. (**B**) and (**C**) Immunostaining indicates that the two steps of magnetic force exposure led to disorganized endothelial actin filaments and disrupted adherens junctions (Yellow arrows indicate the disrupted adherens junctions). (**D**) and (**E**) After overnight recovery without magnetic force, the endothelial actin filaments reorganized and the continuous adherens junctions were restored. In **B** through **E**, red: actin, green: VE-cadherin, white: MNP, and blue: nuclei. Scale bar = 20 μm.

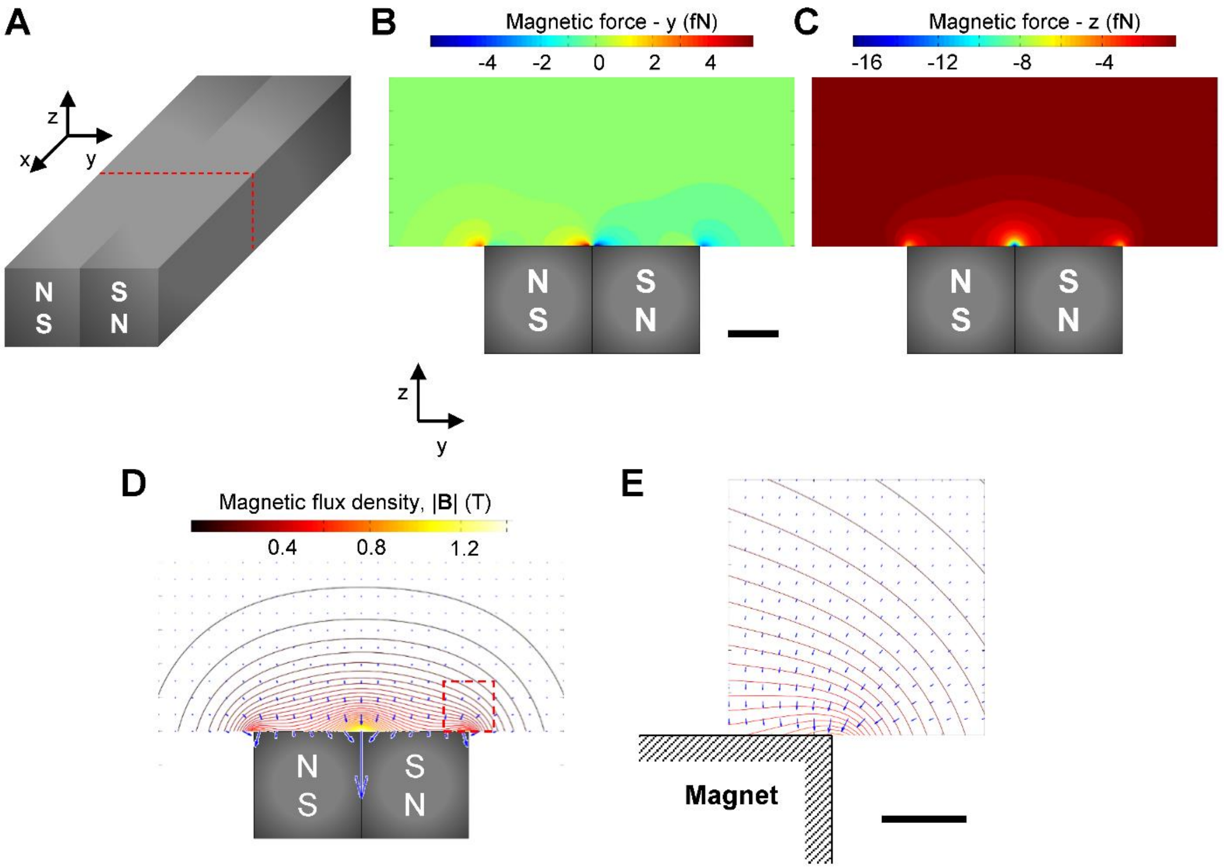

Supplementary Figure 12. The magnetic force field *in vivo*. **A**) Diagram of the magnets (W×H×L = 1/4″×1/4″×1″). **B**) Contour map of magnetic force in the y direction. Scale bar = 3 mm. **C**) Contour map of magnetic force in the z direction. **D**) Overlaid contour lines of magnetic flux density and magnetic force vectors. Dashed square marks the region used for **E**) magnetic treatment of the mouse lateral vein. The force components and vectors within the plane marked with dashed lines in **A**) are plotted. Scale bar = 1 mm.

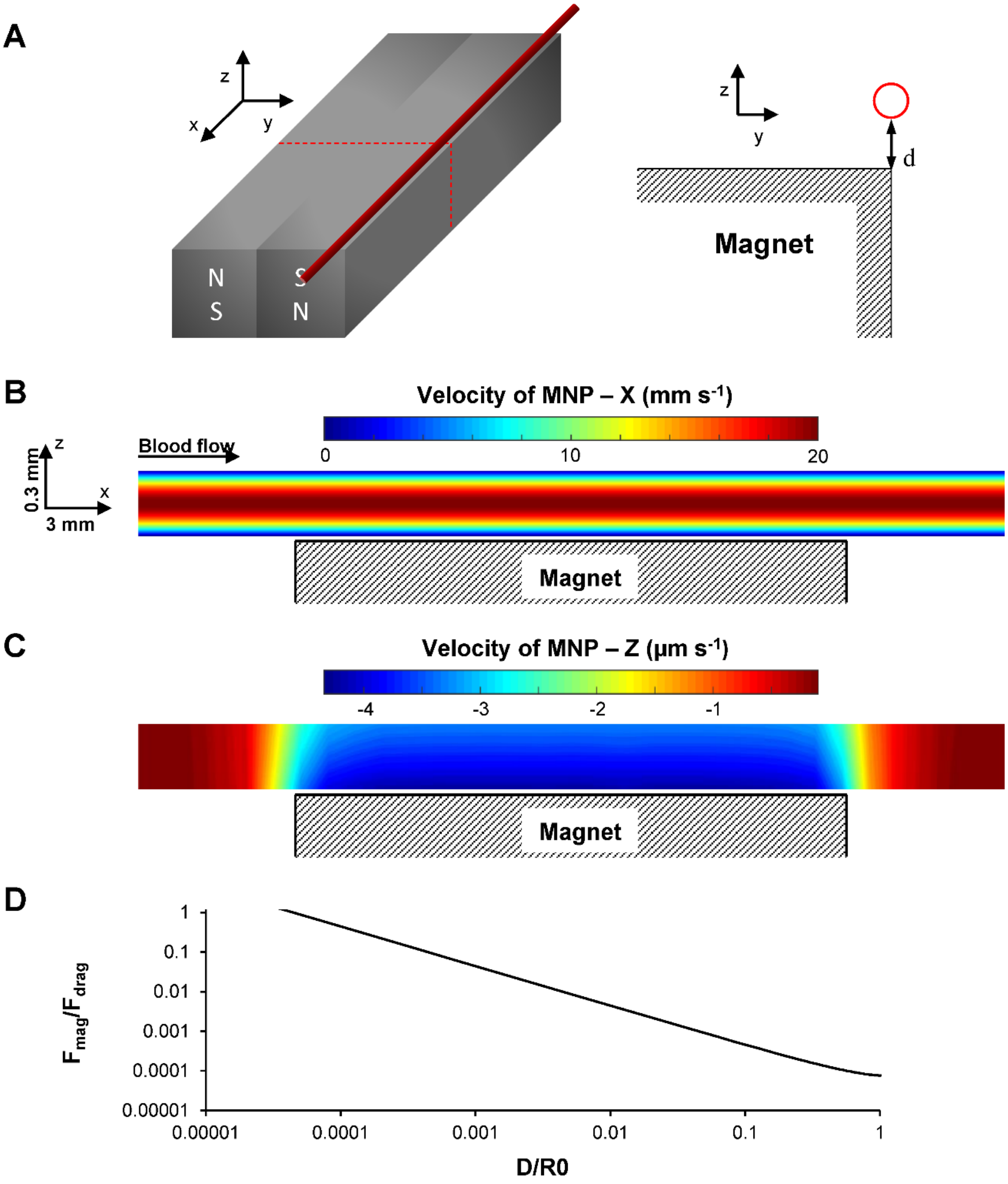

Supplementary Figure 13. Simulated drift Velocity of MNPs in the lateral tail vein. **A**) Model geometry. The distance between the lateral tail vein and the edge of the magnet is assumed to be 1 mm. The diameter of the vein is set at 0.3 mm. **B**) The velocity of MNPs in the x direction. **C**) The velocity of MNPs in the z direction. Note that the aspect ratio between x and z in **B**) and **C**) is 1:10. The gap between the vessel and the magnet is not drawn to proportion. **D**) The ratio between the magnetic force and the fluid drag force along the dashed line in **B**). D is the distance to the bottom vessel wall and R0 is the radius of the vessel.

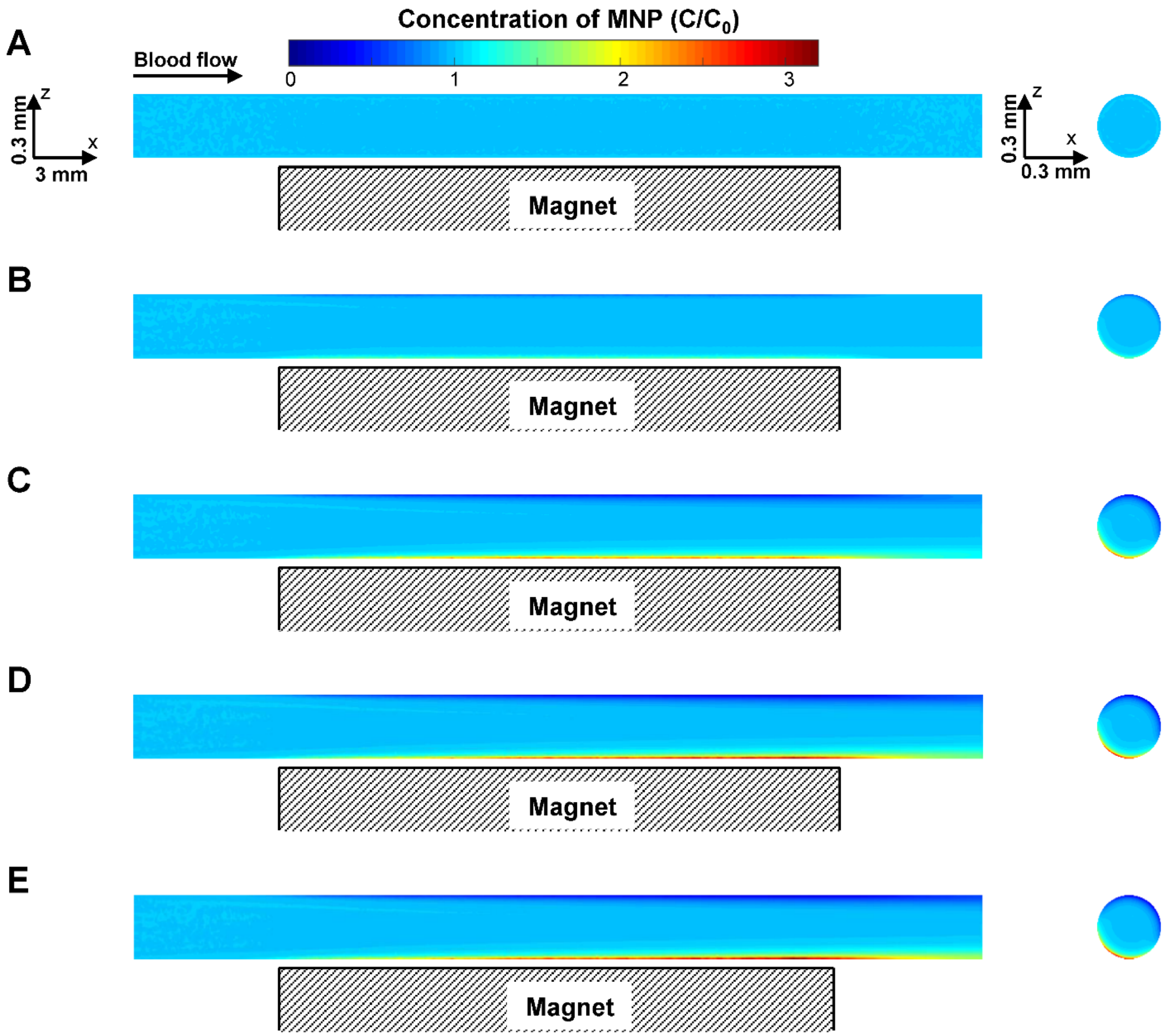

Supplementary Figure 14. The simulated concentration profile of 30 nm MNPs in the lateral tail vein. Above figures show the simulated concentration profiles of MNPs at **A**) 0 s, **B**) 1 s, **C**) 5 s, **D**) 10 s and **E**) 30 s. Note that the aspect ratio between x and z is 1:10. The gap between the vessel and the magnet is not drawn to proportion.

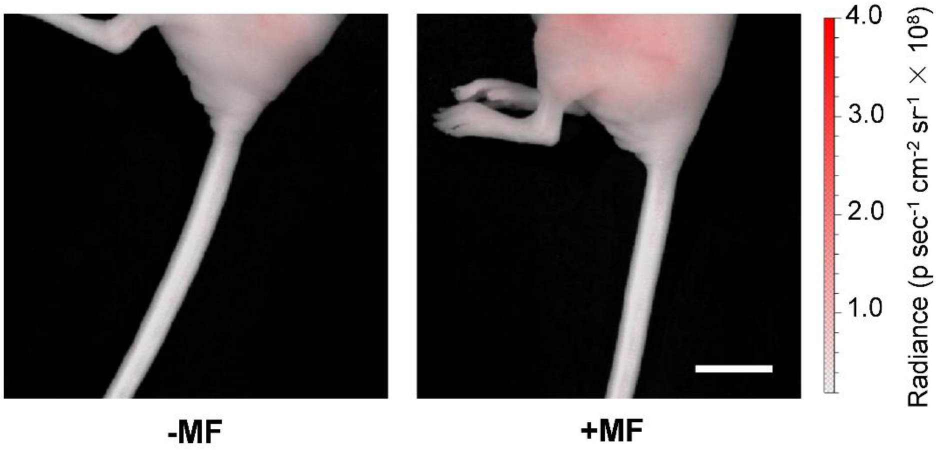

Supplementary Figure 15. Magnetic targeting of 16 nm MNPs. Magnetic force exerted on 16 nm MNPs is insufficient to induce magnetic targeting of 16 nm MNPs in the mouse lateral tail vein. Scale bar = 10 mm.

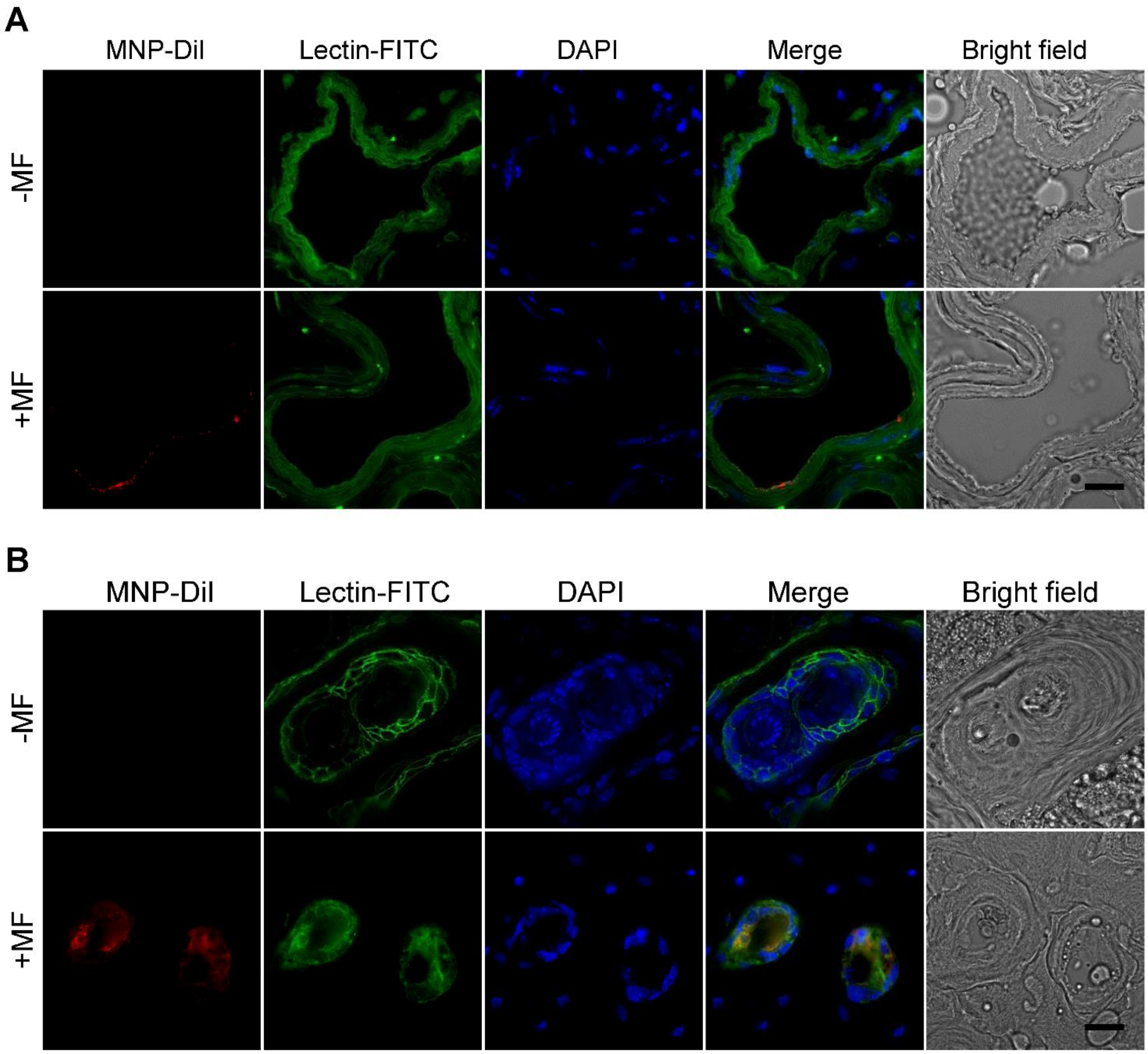

Supplementary Figure 16. Histological examination of MNP distribution in vessels. MNPs only located in the endothelium but not in the interstitial tissues after exposed to the magnetic field as indicated by the histological study of the mouse tail cross sections. **A**) images of larger vein cross sections (top: without magnetic field; bottom: with a magnetic field); **B**) images of smaller vein cross sections (top: without magnetic field; bottom: with a magnetic field). Scale bar = 20 µm.

Supplementary Tables

Supplementary Table 1. Physical properties of MNPs

Core size (nm)	Hydrodynamic diameter (nm)	Polydispersity (%)	Zeta potential (mV)
16	30.1[a]	16.2	-0.94±0.17[b]
33	52.4	11.2	-8.0±0.4

[a] All data are the mean of 3 measurements.

[b] Zeta potential was measured in 0.1 × PBS.

Supplementary Table 2. Lists of numerical simulations

Magnetic flux density	Static cell culture	Supplementary Figure 3
	Microfluidic channel	Supplementary Figure 4
	Lateral tail vein	Supplementary Figure 5
Magnetic force field	Static cell culture	Supplementary Figure 3
	Microfluidic channel	Supplementary Figure 7
	Lateral tail vein	Supplementary Figure 12
Magnetic targeting	Drift velocity	Supplementary Figure 13
	Accumulation of MNPs	Supplementary Figure 14

Supplementary References

1. Pankhurst, Q. A.; Connolly, J.; Jones, S. K.; Dobson, J., Applications of Magnetic Nanoparticles in Biomedicine. *J Phys D Appl Phys* 2003, 36, R167-R181.

2. Furlani, E. P.; Ng, K. C., Nanoscale Magnetic Biotransport with Application to Magnetofection. *Phys Rev E* 2008, 77.
